# Intramolecular Proton Transfer in the Radical Anion of Cytidine Monophosphate Sheds Light on the Sensitivities of Dry vs Wet DNA to Electron Attachment-Induced Damage

**DOI:** 10.1021/jacs.3c00591

**Published:** 2023-04-11

**Authors:** Lidia Chomicz-Mańka, Anna Czaja, Karina Falkiewicz, Magdalena Zdrowowicz, Karol Biernacki, Sebastian Demkowicz, Farhad Izadi, Eugene Arthur-Baidoo, Stephan Denifl, Zhaoguo Zhu, Burak Ahmet Tufekci, Rachel Harris, Kit H. Bowen, Janusz Rak

**Affiliations:** † Laboratory of Biological Sensitizers, Department of Physical Chemistry, Faculty of Chemistry, University of Gdańsk, Wita Stwosza 63, 80-308 Gdańsk, Poland; ‡ Department of Organic Chemistry, Faculty of Chemistry, Gdańsk University of Technology, Narutowicza 11/12, 80-233 Gdańsk, Poland; § Institut für Ionenphysik und Angewandte Physik and Center for Biomolecular Sciences Innsbruck, Leopold-Franzens Universität Innsbruck, Technikerstrasse 25, A-6020 Innsbruck, Austria; ∥ Department of Chemistry, Johns Hopkins University, Baltimore, Maryland 21218, United States

## Abstract

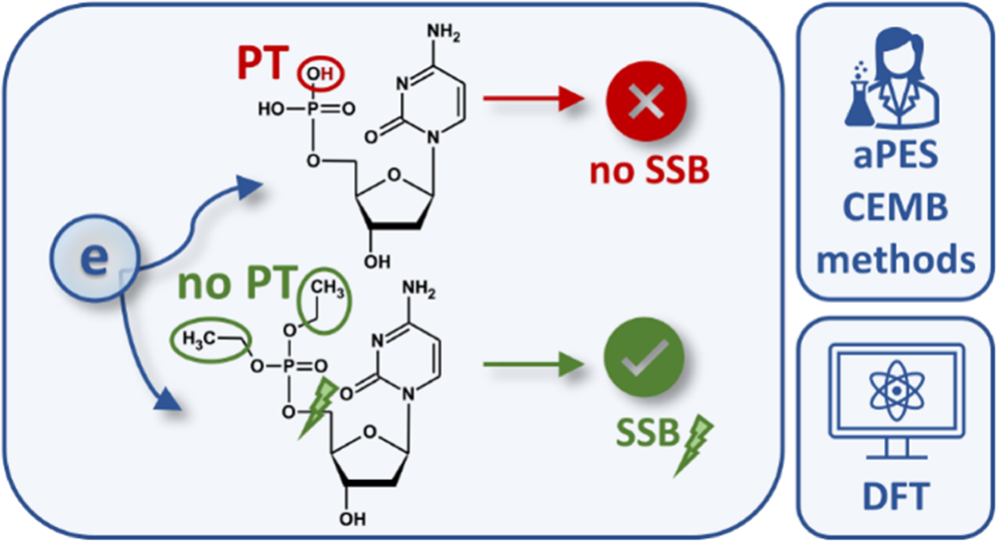

Single-strand breaks (SSBs) induced via electron attachment were previously observed in dry DNA under ultrahigh vacuum (UHV), while hydrated electrons were found not able to induce this DNA damage in an aqueous solution. To explain these findings, crossed electron-molecular beam (CEMB) and anion photoelectron spectroscopy (aPES) experiments coupled to density functional theory (DFT) modeling were used to demonstrate the fundamental importance of proton transfer (PT) in radical anions formed via electron attachment. Three molecular systems were investigated: 5′-monophosphate of 2′-deoxycytidine (dCMPH), where PT in the electron adduct is feasible, and two ethylated derivatives, 5′-diethylphosphate and 3′,5′-tetraethyldiphosphate of 2′-deoxycytidine, where PT is blocked due to substitution of labile protons with the ethyl residues. CEMB and aPES experiments confirmed the cleavage of the C3′/C5′–O bond as the main dissociation channel related to electron attachment in the ethylated derivatives. In the case of dCMPH, however, electron attachment (in the aPES experiments) yielded its parent (intact) radical anion, dCMPH^–^, suggesting that its dissociation was inhibited. The aPES-measured vertical detachment energy of the dCMPH^–^ was found to be 3.27 eV, which agreed with its B3LYP/6-31++G(d,p)-calculated value and implied that electron-induced proton transfer (EIPT) had occurred during electron attachment to the dCMPH model nucleotide. In other words, EIPT, subduing dissociation, appeared to be somewhat protective against SSB. While EIPT is facilitated in solution compared to the dry environment, the above findings are consistent with the stability of DNA against hydrated electron-induced SSB in solution versus free electron-induced SSB formation in dry DNA.

## 1 Introduction

Radiotherapy, one of the most common modalities employed in cancer treatment, eliminates cancerous cells by damaging cellular DNA, making it unlikely that they will replicate.^1^ The detrimental agents are mainly products of water radiolysis, which are produced by sparsely ionizing radiation (X-rays and high-energy electrons are usually used in radiation oncology^2^) that passes through patients′ bodies during radiotherapy.^3^ The most abundant products of water radiolysis are hydroxyl radicals and hydrated electrons.^4^ While the former have long been considered efficient genotoxic agents, the role of hydrated electrons in DNA damage is still puzzling. Early biological experiments carried out in the 1980s^5^ and later^6^ demonstrated that hydrated electrons do not deactivate DNA. However, this paradigm changed around the year 2000 due to the seminal work of Leon Sanche. His group measured resonance curves that showed the formation of both single (SSBs) and double (DBSs) strand breaks in the plasmid DNA under ultrahigh vacuum due to bombardment by low-energy electrons, i.e., due to electrons with sub-DNA ionization threshold energies.^7^ This discovery resulted in a flood of papers, both experimental^8−16^ and theoretical,^17−23^ that tackled the problem of destructive interactions between electrons and DNA as well as its building blocks: nucleosides, nucleotides, nucleobases, and deoxyribose. To this end, various joint theoretical-experimental reports were published by the collaborating groups of Rak, Gutowski, and Bowen.^24^ These studies combined anion photoelectron spectroscopy (aPES) with density functional theory (DFT) modeling to interpret the measured photoelectron spectra of DNA component-containing anionic complexes in terms of barrier-free (electron-induced) proton transfer; these processes are thus called barrier-free proton transfer (BFPT) or EIPT. Stable valence anions rather than resonances were also suggested to be responsible for the C3′–O and C5′–O bond breakages due to electron attachment to the nucleotides of cytosine and thymine.^20,21^ On the other hand, Simons^17^ suggested that DNA SSB is a result of shape resonance formation on a nucleobase. This resonance is then coupled to electron transfer from the π* orbital localized on the nucleobase to the σ* orbital of the C*X*′–O bond, where X = 3 or 5. Such an electron-transfer process leads directly to the cleavage of the phosphate bond—a hallmark of SSB in DNA.

However, it is worth emphasizing that some radiation chemistry papers published after 2000 demonstrated no SSB formation in an aqueous solution during DNA radiolysis when the hydroxyl radicals were scavenged. Indeed, in a radiation chemical study devoted to the 5-bromo-2′-deoxyuridine (BrdU)-labeled oligonucleotides, Sanche et al.^25^ showed that strand breakages are not formed in the native, nonlabeled oligonucleotides when hydroxyl radicals are scavenged during γ-irradiation even for doses as large as 700 Gy (for comparison, in fractionated radiotherapy, the radiation doses do not exceed 2 Gy per session). Similarly, Rak et al.^26^ irradiated with 140 Gy (γ-rays) the solutions of 5′-TXT single-stranded trinucleotides, where X = T, C, A, or G, in the presence of *tert*-butanol as the hydroxyl radical scavenger. With high-performance liquid chromatography (HPLC) analysis following immediately after the irradiations, no traces of SSBs were detected. Only a small amount of dihydropyrimidine in 5′-TCT and 5′-TTT trimers was confirmed with the liquid chromatography/mass spectrometry (LC/MS) method. As opposed to native sequences, SSBs (and other types of damages) were observed under the same irradiation conditions only when the middle nucleobase in the studied trimers was substituted with its brominated analogue.

The striking difference in reactivity toward excess electrons of DNA under ultrahigh vacuum and in an aqueous solution might be related to proton transfer involving the initial radical anion of a nucleobase formed in the electron attachment process. Such a suggestion was examined in an ab initio molecular dynamics study devoted to aqueous solutions of DNA nucleotides published by Kohanoff et al.^27^ Namely, for the 3′-phosphates of native nucleosides, they demonstrated that protonation by water significantly increases the barriers and/or thermodynamic stimuli to the phosphate-bond dissociation induced by hydrated electron attachment. Thus, their findings showed that protonation may prevent SSB occurrence triggered by the attachment of electrons in hydrated DNA. To this end, it is worth mentioning that proton transfer from a complementary base to the negative ion resonant state formed on the second base in the nucleobase pair may be responsible for the excess electron autodetachment, thus preventing DNA damage.^28^

**Scheme 1:**
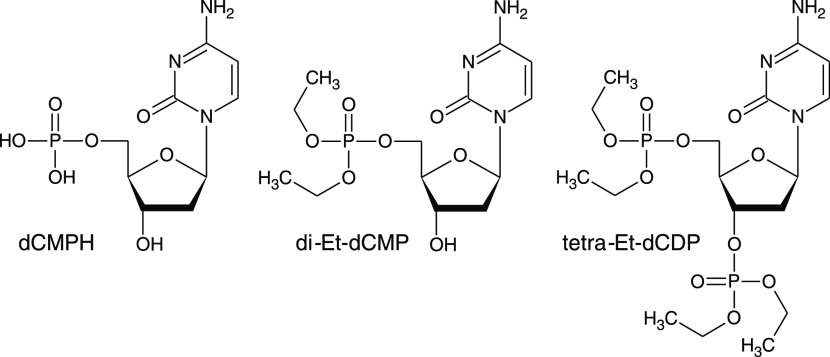
Structures of Cytosine 5′-Monophosphate (dCMPH), Ethyl Ester of Cytosine 5′-Monophosphate (di-Et-dCMP), and Ethyl Ester of Cytosine 3′,5′-Diphosphate (tetra-Et-dCDP)

In the current paper, we investigate the paramount role of proton transfer, triggered by electron attachment to molecules of biological relevance. Namely, we analyze dissociative electron attachment (DEA) in the gas phase to the exemplar pyrimidine nucleoside, 2′-deoxycytosine 5′-monophosphate (dCMPH), and two phosphates of 2′-deoxycytosine substituted with the ethyl groups to prevent possible proton transfer: ethyl ester of cytosine 5′-monophosphate (di-Et-dCMP) and ethyl ester of cytosine 3′,5′-diphosphate (tetra-Et-dCDP). The corresponding molecular structures are shown in Scheme 1. We combine DFT modeling with crossed electron-molecular beam (CEMB) experiments and anion photoelectron spectroscopy (aPES) to demonstrate the influence of intramolecular proton transfer on the electron attachment-induced degradation of the studied nucleotides. We also discuss possible implications for SSB formation in DNA upon low-energy electron attachment.

## 2 Methods

### 2.1 Experimental Section

#### 2.1.1 Synthesis

**Scheme 2:**
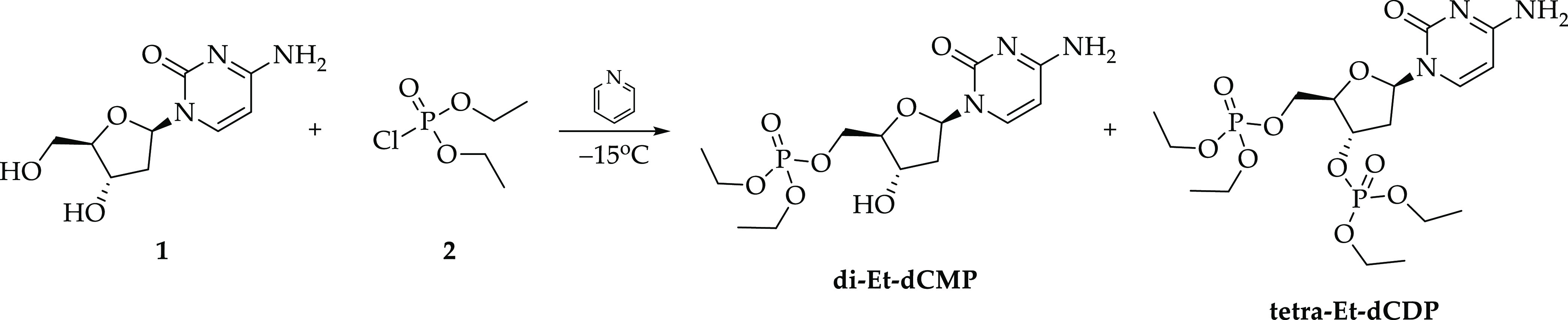
Synthesis of di-Et-dCMP and tetra-Et-dCDP

*O*,*O*-Diethyl-5′-deoxycytidine monophosphate (di-Et-dCMP) and *O*,*O*,*O*,*O*-tetraethyl-3′,5′-deoxycytidine diphosphate (tetra-Et-dCDP) were synthesized according to the procedure described in Scheme 2.

Under anhydrous conditions, **1** (1 equiv, *n* = 0.0088 mol, *m* = 2 g) was suspended in 40 mL of dry pyridine in a burner-dried round-bottom flask, and then, the mixture was cooled down to −15°C, followed by dropwise addition of **2** (2 equiv, *n* = 0.0176 mol, *m* = 3.038 g). The obtained suspension was slowly warmed up to room temperature and then left for an additional 20 min with stirring. Next, the reaction mixture was evaporated under vacuum, and the subsequently obtained crude, sticky, yellowish oil was prepurified with column chromatography using CHCl_3_/MeOH (20:1) as the eluent. The final products were purified with semipreparative HPLC (Shimadzu, LC 20AD, Kyoto, Japan) equipped with a ultraviolet (UV) detector (SPD M20A), which was set at 283 nm. For the separation, the Synergy Polar-RP C18 column (Phenomenex, Gemini, Warsaw, Poland; 150 mm × 10 mm, 5 μL in particle size and 110 Å in pore size) was used, at 25 °C with a flow rate of 4 mL/min. The linear gradient of 0–80% phase B in 10 min was used (mobile phase A: 0.1% HCOOH in water, B: 80% acetonitrile in water). Products were obtained as colorless, sticky oils. Tetra-Et-dCDP was obtained with 25% yield (*m* = 1.118 g, *n* = 0.00224 mol) and di-Et-dCMP with 44% yield (*m* = 1.401 g, *n* = 0.00386 mol). 2′-Deoxycytidine **1**, diethyl phosphorochloridate **2**, and anhydrous pyridine were purchased from Merck.

Identification of di-Et-dCMP: ^1^H NMR (500 MHz, CD_3_OD, Figure S1a) δ_H_ 8.10 (d, *J* = 7.9 Hz, 1H), 6.19 (t, *J* = 6.3 Hz, 1H), 6.14 (d, *J* = 7.9 Hz, 1H), 4.43–4.37 (m, 1H), 4.34–4.28 (m, 1H), 4.28–4.21 (m, 1H), 4.20–4.11 (m, 5H), 2.45 (ddd, *J* = 13.9, 6.4, 4.2 Hz, 1H), 2.28 (dt, *J* = 13.4, 6.4 Hz, 1H), 1.42–1.29 (m, 6H); ^31^P NMR (202 MHz, CD_3_OD, Figure S2a) δ_P_ −1.38; ^13^C NMR (125 MHz, CD_3_OD, Figure S3a) δ_C_ 159.8 (s), 146.9 (s), 144.6 (s), 93.3 (s), 87.0 (s), 85.6 (d, *J* = 7.5 Hz), 70.0 (s), 66.7 (d, *J* = 5.9 Hz), 64.4 (d, *J* = 1.3 Hz), 64.3 (d, *J* = 1.7 Hz), 39.8 (s), 15.1 (d, *J* = 1.0 Hz), 15.0 (d, *J* = 1.4 Hz); high resolution MS (HRMS) (Figure S4a) calculated for C_13_H_22_N_3_O_7_P: 362.1123 [M – H]^−^; found: 362.1247; UV–vis spectrum (water; Figure S5a), λ_max_ = 279 nm.

Identification of tetra-Et-dCDP: ^1^H NMR (500 MHz, DMSO-*d*_6_, Figure S1b) δ_H_ 7.62 (d, *J* = 7.5 Hz, 1H), 7.25 (d, *J* = 14.1 Hz, 2H), 6.17 (dd, *J* = 7.4, 6.5 Hz, 1H), 5.74 (d, *J* = 7.5 Hz, 1H), 4.94–4.88 (m, 1H), 4.23–4.20 (m, 1H), 4.20–4.16 (m, 1H), 4.13 (dd, *J* = 11.5, 5.9 Hz, 1H), 4.10–4.00 (m, 8H), 2.44 (ddd, *J* = 14.1, 6.1, 2.8 Hz, 1H), 2.28 (dt, *J* = 14.0, 7.0 Hz, 1H), 1.29–1.22 (m, 12H); ^31^P NMR (202 MHz, DMSO-*d*_6_, Figure S2b) δ_P_ −1.15 (s), −2.03 (s); ^13^C NMR (125 MHz, DMSO-*d*_6_, Figure S3b) δ_C_ 166.1 (s), 164.2 (s), 155.2 (s), 141.5 (s), 94.8 (s), 85.9 (s), 83.0 (t, *J* = 7.4 Hz), 77.2 (d, *J* = 5.3 Hz), 66.5 (d, *J* = 5.3 Hz), 64.2 (d, *J* = 3.8 Hz), 64.1 (d, *J* = 3.6 Hz), 64.0 (d, *J* = 2.6 Hz), 63.9 (d, *J* = 3.0 Hz), 38.0 (d, *J* = 4.0 Hz), 16.4 (d, *J* = 6.5 Hz); HRMS (Figure S4b) calculated for C_17_H_31_N_3_O_10_P_2_: 498.1412 [M – H]^−^; found: 498.2049; UV–vis spectrum (water; Figure S5b), λ_max_ = 278 nm.

NMR spectra were recorded on Varian Unity Inova 500 MHz. Chemical shifts are reported in parts per million (ppm), relative to the residual signal of DMSO-*d*_6_ (2.50 ppm). The MS measurements (electrospray ionization; negative mode, spray voltage −4.5 kV; source temperature 300 °C) were performed with the use of a TripleTOF 5600^+^ (SCIEX, Framingham, MA). Ultra HPLC (UHPLC) chromatograms and UV–vis spectra were recorded on a Nexera X2 system (Shimadzu, Kyoto, Japan) with a diode array detector (Kinetex column, Phenomenex, 1.7 μm, C18, 100 Å, 2.1 mm × 150 mm; the linear gradient of 0–50% phase B in 20 min; mobile phases: A, 0.1% HCOOH in water; B, 80% acetonitrile in water; flow 0.3 mL/min; oven temperature 25 °C).

#### 2.1.2 Crossed Electron-Molecule Beam (CEMB) Experiments

The CEMB apparatus in Innsbruck was used to collect the DEA data in the gas phase. The setup has been described in detail in our previous work,^29^ and only a brief description in relation to the current study is provided. A hemispherical electron monochromator (HEM) serves as an ionization source. It provides electrons with a narrow energy distribution of the Gaussian profile (∼100 meV at full width at half-maximum). The incident electron current was 20–40 nA, which was measured after the interaction region with the neutral beam by a Faraday plate. The acquired current was measured with a picoammeter. By applying the appropriate electric potentials for the lens stack after the HEM briefly before the interaction region, the energy of the electron beam was set. As mentioned above, di-Et-dCMP and tetra-Et-dCDP appear as liquid at room temperature. The sample vapor was transferred from the sample container into the vacuum chamber via an external gas inlet coupled with a precision valve in experimental setups as used in.^30^ The sample vapor entered the interaction region of the HEM via a 1 mm stainless-steel capillary. To obtain enough vapor for the experiment, the samples were heated in the container. A thermocouple temperature sensor was used to measure the temperature of the effusive molecular beam source. The typical temperatures used for di-Et-dCMP and tetra-Et-dCDP were 360 and 364 K, respectively.

A weak electrostatic field was used to extract the negatively charged ions formed into a quadrupole mass spectrometer (QMS). For low collision energies, CEMB experiments at which the key anions discussed in the text were formed, no excited states are involved. The quadrupole used for mass analysis has a nominal mass range of 2048 u. Finally, the mass-separated anions were detected using a channeltron-type secondary electron multiplier set to single pulse counting. The ion yield curves were obtained by measuring the intensity of a given mass-separated anion as a function of incident electron energy. The experiments were carried out at working pressures of 4.8 × 10^–8^ and 1.3 × 10^–7^ mbar for di-Et-dCMP and tetra-Et-dCDP, respectively (10^–8^ mbar background pressure).

In addition to the studies with esters, we investigated electron attachment to the native dCMPH sample (powder, ≥95%), which was purchased from Sigma Aldrich (Vienna, Austria) and used as delivered. For this sample, a copper furnace with a glass inset was used as a sample container. Vaporized molecules were guided to the interaction region by a capillary with a diameter of 1 mm. The oven had to be heated to temperatures approximately between 388 and 398 K to achieve a signal of negative ions. However, measurements of the electron ionization mass spectra at different lower temperatures indicated varying ion intensities as well as an onset of sample polymerization at 391 K. In addition, a burned residual was present in the glass inset when the oven was opened after the measurements. Therefore, it can be supposed that a part of the sample degraded during the heating process. The working pressure for this sample was 1.1 × 10^–7^ mbar.

In order to calibrate the ion yield curves of the negative ions, we used the well-known 0 eV resonance for the formation of Cl^–^/CCl_4_ arising from s-wave electron attachment processes.^31^

#### 2.1.3 Photoelectron Spectroscopy (PES) Measurements

Anion photoelectron spectroscopy is conducted by crossing a mass-selected beam of negative ions with fixed-frequency photons, followed by energy analysis of the resulting photodetached electrons. Photodetachment is governed by the energy-conserving relationship, *h*ν = EBE + EKE, where *h*ν is the photon energy, EBE is the electron binding energy, and EKE is the electron kinetic energy. Our anion photoelectron spectrometer has been described in detail previously.^32^

The parent anions of dCMPH were generated using a novel pulsed infrared desorption-pulsed visible photoemission anion source.^33^ In the first stage of the experiment, low-power infrared laser pulses (1.17 eV/photon) from a neodymium-doped yttrium aluminum garnet (Nd:YAG) laser were used to desorb neutral dCMPH from a slowly translating graphite bar, which was thinly coated with the sample. Simultaneously, electrons were generated by visible laser pulses (another Nd:YAG laser operated at 532 nm, 2.33 eV/photon) striking a yttrium wire. At the same time, 100 psig of pure helium gas was expanded over the graphite bar and metal wire by a pulsed valve, providing a collisionally cooled jet to carry away excess energy and stabilize the resulting parent anions. Thus, any chemical reaction triggered in our spectrometer follows excited-state pathways. The photoelectron spectrum was recorded by crossing a mass-selected beam of parent anions (using a time of flight (TOF) mass spectrometer internal to the larger aPES apparatus) with a fixed-frequency photon beam (a third Nd:YAG laser operated at 355 nm, 3.49 eV/photon). The photodetached electrons were energy-analyzed using a magnetic bottle energy analyzer with a resolution of 50 meV at EKE = 1 eV. The photoelectron spectrum was calibrated against the well-known photoelectron spectrum of Cu^–^.^34^

### 2.2 Computational Details

Electron attachment to dCMPH in the gas phase was studied as in the Kobyłecka et al. report^35^ at the density functional theory level, with the use of the B3LYP functional^36^ and the 6-31++G(d,p) basis set.^37−39^ The dCMPH *south*-*syn* conformer was used as a starting structure for generating the remaining systems. This type of sugar ring conformation was observed for 2′-deoxyadenosine 5′-monophosphate (dAMPH) as favoring the BFPT process.^34^ The *south*-*syn* conformation could potentially support the proton transfer process between the phosphate group and the O2 atom attached to the pyrimidine ring in dCMPH. Additionally, the conformer *south*-*anti* was analyzed too as it could potentially support proton transfer from the phosphate group to the C5 or C6 positions of the pyrimidine ring.

Electron attachment-induced degradation of di-Et-dCMP and tetra-Et-dCDP esters was analyzed at the same theory level (B3LYP/6-31++G(d,p), gas phase). The free enthalpy change concerning the formation of the product complex is denoted Δ*G*, while Δ*G*_sep_ stands for the free enthalpy change of the process in which anionic and radical products are calculated separately, i.e., they are separated to infinity. ΔG* indicates the activation barrier in the Gibbs free energy scale. The AEA_G_ (adiabatic electron affinity) was calculated as the Gibbs free energy difference between the neutral and its corresponding anion radical, both in their optimum geometries.

In order to estimate the position of a chosen resonance, a stabilization method was employed.^40^ Namely, in order to artificially stabilize the anionic resonance state, a fractional amount of the nuclear charge (Δ*q*) at pyrimidine ring atomic centers was increased. Then, the electron binding energy, *D*, was calculated as a difference between the energy of the neutral and that of the anion radical. Extrapolation of the binding energy, *D*, to Δ*q* equal to zero enables the resonance energy (*E*_R_), i.e., the position of a resonance, to be estimated. All resonance calculations were conducted at the MP2/cc-pvdz level, which occurred to have the best quality-to-time properties in our previous resonance studies.^41^

The thermodynamic thresholds of DEA reactions at the standard (298.15 K) and experimental (360.95 K) temperatures were calculated for the most stable conformers of dCMPH, di-Et-dCMP, and tetra-Et-dCDP as the difference between the enthalpies of reactants in their ground state. The geometries of reactants were optimized at the M06-2X^42^/aug-cc-pVTZ^43^ level of theory based on the geometries obtained at the B3LYP/6-31++G(d,p) level. This approach was previously shown to be successful.^44,45^

All of the calculations were carried out with the Gaussian16 suite of programs.^46^

## 3 Results and Discussion

### 3.1 Molecular Models

**Figure 1:**
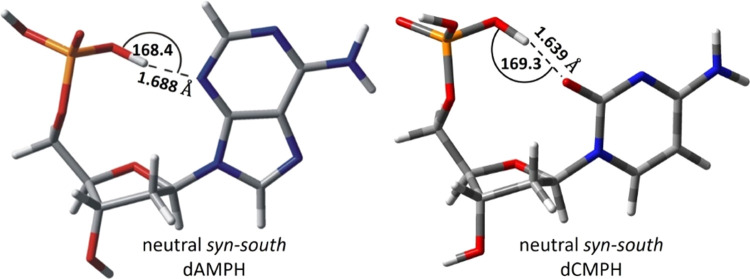
3D visualization of the most stable neutral conformations of 2′-deoxyadenosine 5′-monophosphate (dAMPH)^34^ and 2′-deoxycytidine 5′-monophosphate (dCMPH). Structure of dAMPH reprinted from ref (34), with the permission of AIP Publishing.

A striking difference between the effect of interactions between electrons and DNA in the gas phase and aqueous solution might be produced by proton transfer (PT) possible only in water. If proton transfer was a main factor that hinders SSB in DNA, it should be possible to demonstrate, using appropriate molecular models, that PT does stop the breakage of the phosphoester bond in a DNA-like species. The smallest system that represents the DNA structure and could, in principle, undergo phosphoester bond dissociation is a nucleotide. Of course, such a model is not able to describe the entire process of electron interactions with DNA. One should remember, however, that the excess electron is strongly localized to particular bases in the π-stack since charge localization is simply governed by electron affinities of interacting nucleobases,^47^ DNA conformational effects on charge distribution are quite small,^47^ and excess electron-transfer couplings were found to be considerably smaller than the corresponding couplings for hole transfer,^48^ which additionally implies that long-range ET is possible only for very specific sequences and, therefore, quite limited for a random DNA. The above-mentioned facts suggest that electron attachment-induced DNA damage is rather a local phenomenon. Therefore, our model, which is limited to a nucleotide, seems to be sufficient to describe strand break formation as it contains the main elements of the studied process. Indeed, it possesses the phosphoester bond, has positive electron affinity^49^ corresponding to electron attachment to the π* orbital localized on the nucleobase,^46^ and in such a system the excess electron can move from the initial π* state to the σ* orbital of the C*X*′–O (where *X*′ = 3′ or 5′) bond, resulting in its breakage (an equivalent of SSB). To this end, it is worth noticing that several years ago we published back-to-back papers employing photoelectron spectroscopy of negative anions^50^ and DFT calculations,^34^ which demonstrate that electron attachment to dAMPH is primarily followed by an intramolecular proton transfer between the phosphate and the adenine residue to form a thermodynamically stable radical anion. A similar situation might be observed in pyrimidine nucleotides. Purine nucleobases possess two condensed rings, six- and five-membered, which suggest stereochemical closeness of the phosphate group (proton donor) and proton acceptor centers of a nucleobase in the purine nucleotide. On the other hand, pyrimidine nucleotides comprise only one six-member ring. Nevertheless, the internuclear distances in a representative conformation of pyrimidine nucleotides imply the possibility of intranucleotide PT as well (cf. the respective interatomic distances shown in Figure 1 for dAMPH and dCMPH, respectively). Consequently, dCMPH could be considered a model system in which electron attachment-induced PT (EIPT) may occur in the gas phase and block the “strand break”. This process would lead to the formation of the parent anion. On the other hand, if the labile protons are substituted with the alkyl groups, EIPT is no longer possible and the primary anions should dissociate, resulting in the cleavage of the C*X*′–O bond. In the following, we will show the results of CEMB and PES experiments combined with quantum chemical analysis on dCMPH and two ethylated cytidine phosphates (see Scheme 1), which strongly support our hypothesis about the paramount role of EIPT in the formation of the C–O bond breakage.

### 3.2 Electron-Molecule Beam Experiments

The CEMB experiment allows one to identify the main channels associated with the degradation of a molecular system interacting with an electron of defined energy. Thus, these experiments seem to be ideally suited for the verification of the formulated above hypothesis. Indeed, comparison of the detected anionic species for dCMPH with those for di-Et-dCMP and tetra-Et-dCDP should prove or discard the involvement of PT in the degradation of the studied anionic systems.

**Figure 2:**
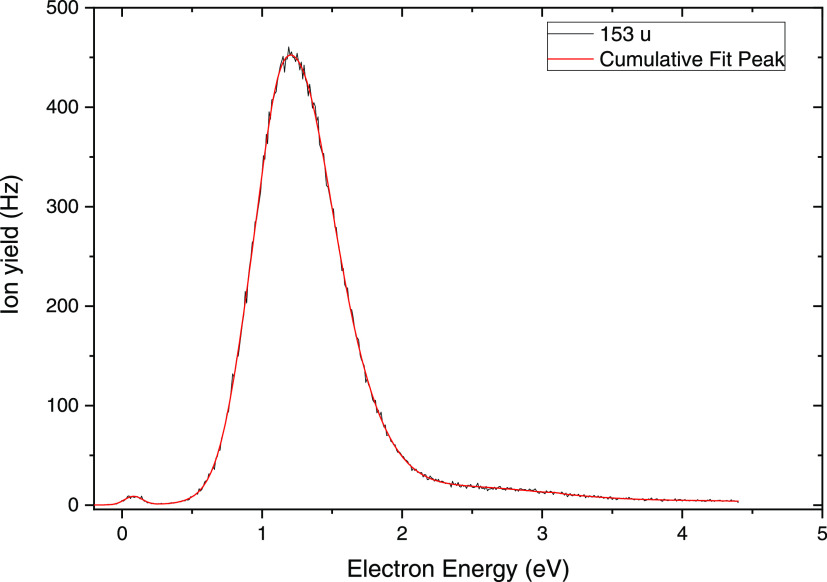
Anion efficiency curve for the formation of C_4_H_10_PO_4_^–^ upon electron attachment to di-Et-dCMP. Black line, experimental data; red line, cumulative peak fit.

In our electron attachment study with the di-Et-dCMP, five anionic species with *m*/*z* ratios of 288 (C_9_H_11_N_3_O_6_P^–^), 207 (C_8_H_16_O_4_P^–^), 153 (C_4_H_10_PO_4_^–^), 43 (C_2_H_3_O^–^), and 17 (OH^–^) were observed. Herein, we place our emphasis on the anion at *m*/*z* 153 C_4_H_10_PO_4_^–^, which was also the most abundant anion formed. Hence, we show only the anion efficiency curve of C_4_H_10_PO_4_^–^ in Figure 2. The anion yield curves for the other fragment anions, being at least 2 orders of magnitude weaker in intensity than C_4_H_10_PO_4_^–^, are presented in the Supporting Information (SI, Figure S6). The SI also includes a detailed table on the peak positions derived by multiple peak fitting and the derived experimental and calculated thermodynamic thresholds for all fragment anions formed by electron attachment to di-Et-dCMP (see Table S1). No parent anion of the di-Et-dCMP was observed within the detection limit of the apparatus, which supports the EIPT hypothesis. Indeed, the protons present in the dCMPH molecule are substituted with the ethyl groups in di-Et-dCMP, which disables PT. On the other hand, a large thermodynamic stimulus (reaction enthalpy; Δ*H*) of −18.6 kcal/mol (calculated at the B3LYP/6-31++G(d,p) level) for the release of the ethylated phosphate anion, associated with the activation barrier (enthalpy of activation; ΔH*) of 14.6 kcal/mol, justifies the high yield of the C_4_H_10_PO_4_^–^ anion (see Figure 2).

As shown in Figure 2, C_4_H_10_PO_4_^–^ exhibits a main resonance below the electron energy of 2 eV, with the highest intensity observed at about 1.1 eV. In the tail of the peak at higher energies, peak contributions may span up to a broad band near 2.5 eV. We also observe a weakly abundant peak at 0.05 eV, which may result from DEA to vibrationally excited neutrals. With the use of the stabilization method (see Section 2), we estimated the position (*E*_R_ = 1.64 eV) of the shape resonance localized to the π* orbital at the pyrimidine ring, which after electron transfer to the σ* orbital can be responsible for the experimentally observed C_4_H_10_PO_4_^–^ resonance (SI, Figure S7). The value of 1.64 eV remains in reasonable accordance with the experimental position of this resonance (Figure 2).

C_4_H_10_PO_4_^–^ forms upon the C–O phosphoester bond cleavage within the temporary negative ion. The observed bond cleavage at the electron energy around 1.1 eV is in line with the hypothesis by Simons that single-strand breaks (SSBs) may be induced by initial low-energy electron attachment to the nucleobases^,^^17^ and thus supports the results by Sanche et al. on electron-induced SSB formation in DNA under the UHV environment.^51^ A direct attachment of the electron to the P = O π* orbital leading to the break of the phosphoester bond (model of SSB) is also possible but requires electrons of energies larger than 2 eV, as indicated by respective resonance anion profiles obtained by the charge stabilization method.^17^

In the case of electron attachment to tetra-Et-dCDP, we observed the generation of seven anionic species with *m*/*z* ratios of 261 (C_7_H_8_N_3_O_6_P^–^), 153 (C_4_H_10_PO_4_^–^), 121 (C_6_H_5_N_2_O^–^), 98 (C_5_H_6_O_2_^–^), 59 (C_2_H_3_O_2_^–^), 45 (C_2_H_5_O^–^), and 17 (OH^–^). No parent anion was detectable for the same reason as explained for di-Et-dCDP. The abundance of C_4_H_10_PO_4_^–^ relative to the other fragment anions is even higher than for di-Et-dCDP. Hence, we focus here on the anion formed at *m*/*z* 153, and all information for the other fragment anions is summarized in the SI, including the anion efficiency curve (see Figure S8) and a detailed table of peak positions as well as experimental and theoretical thresholds (see Table S2).

**Figure 3:**
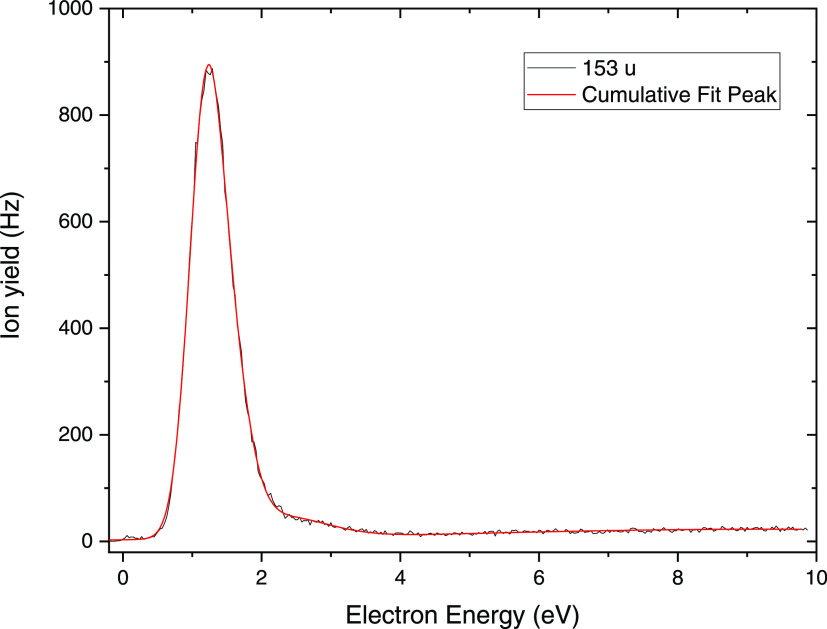
Anion efficiency curve for the formation of C_4_H_10_PO_4_^–^ upon electron attachment to tetra-Et-dCDP. Black line, experimental data; red line, cumulative peak fit.

Figure 3 shows the anion efficiency curve of C_4_H_10_PO_4_^–^ observed for tetra-Et-dCDP in the electron energy range of about 0–10 eV. Again, the curve shows the maximum intensity in the region between 0 and 2 eV and is dominated by a resonance near 1.2 eV. The ion yield is thus very similar to that of C_4_H_10_PO_4_^–^ from di-Et-dCMP. The curve also shows the presence of other resonances along the tail and a minor feature at 0.06 eV (like for di-Et-dCMP, also here ascribed to the initial vibrational excitation). Also, for the tetra-Et-dCDP ester, the formation of C_4_H_10_PO_4_^–^ corresponds to dissociation of a single C–O bond, representing a cleavage related to an SSB in DNA. A simple argument for the stronger relative abundance than in di-Et-dCMP would be the availability of two sites of C_4_H_10_PO_4_^–^ formation in the tetra-Et-dCDP molecule.

Similar to di-Et-dCMP, for tetra-Et-dCDP, the calculated position of the shape resonance (*E*_R_ = 1.57 eV) localized to the second π* orbital at the pyrimidine ring agrees with the experimental maximum for the release of the C_4_H_10_PO_4_^–^ anion (compare Figures 3 and S7). This result supports the electron-transfer mechanism from the nucleobase moiety to the phosphate group. We also note that the calculated reaction enthalpy for C_4_H_10_PO_4_^–^ formation is <−0.15 eV, i.e., no intense resonance near 0 eV (or bound state) seems to lead to the cleavage.

**Figure 4:**
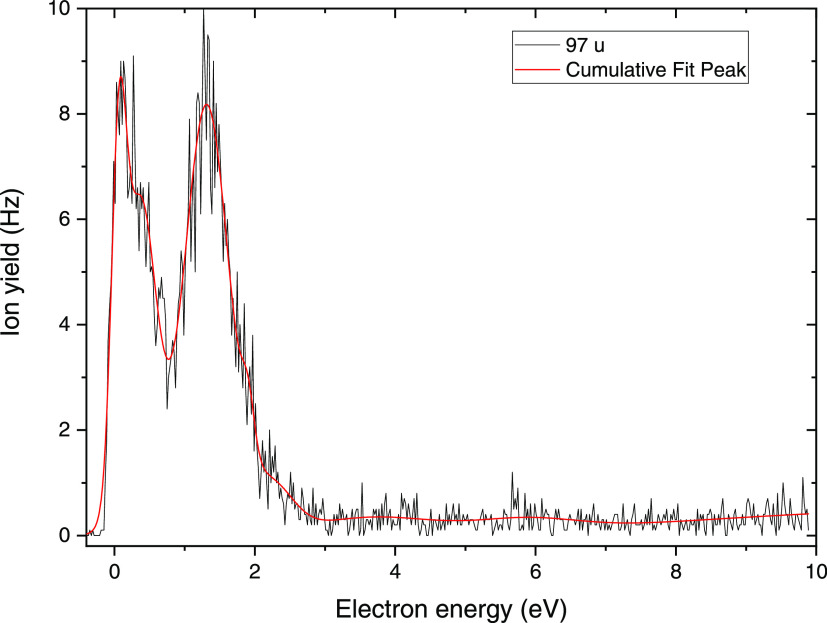
Anion efficiency curve for the production of H_2_PO_4_^–^ via electron attachment to native dCMPH. Black line, experimental data; red line, cumulative peak fit.

In addition to the esters, we also investigated electron attachment to native dCMPH in order to compare a set of data recorded with the same apparatus for the molecular systems where PT is not possible (di-Et-dCMP and tetra-Et-dCDP). However, as pointed out in the Section 2, negative ion data was only obtainable at experimental conditions, where thermal decomposition was likely. All results for this molecule are summarized in the tabular form in the SI (see Table S3). To our surprise, no parent anion was observable. According to the EIPT hypothesis, PT should hinder degradation and EIPT seems to be probable in dCMPH. Indeed, our B3LYP/6-31G++(d,p) calculations predict a thermodynamic stimulus for PT within the anionic dCMPH between −18 and −27 kcal/mol, depending on the site to which a proton is transferred (see Figure 7). However, the main fragment anion observed for this molecule was found at *m*/*z* 97, which may be attributed again to H_2_PO_4_^–^, the phosphate anion. The anion efficiency curve for H_2_PO_4_^–^ is shown in Figure 4 (the efficiency curves for the remaining low-intensity anionic fragments are shown in Figure S9). The ion yield indicates the formation of two main resonances within the electron energy range of 0–2 eV with almost equal intensity. The first maximum is found at ∼0 eV, and the second peak is detectable at around 1.3 eV. A multiple peak fit also indicated minor features at ∼0.3 eV and within the tail of the second peak.

Here, the calculated position of the shape resonance, *E*_R_ = 1.34 eV, localized to the second π* orbital at the pyrimidine ring and corresponding, after electron transfer to the C5–O σ* orbital, to the release of H_2_PO_4_^–^ agrees very well with the experimental maximum of 1.3 eV (compare Figures 4 and S7). In contrast, the ion yield close to 0 eV may be ascribed to attachment to thermally decomposed dCMPH. However, we note that a previous DEA study with d-ribose-5-phosphate (a model compound for the backbone) reported H_2_PO_4_^–^ formation exclusively near 0 eV.^52^ In that study, laser-induced acoustic desorption was used to generate the neutral beam of molecules and thus thermal decomposition should not play a role. The absence of a feature at electron energies >1 eV in the H_2_PO_4_^–^ ion yield from d-ribose-5-phosphate supports the electron-transfer model suggested for dCMPH; on the other hand, the observation of ion yield near 0 eV would also mean that a low-lying resonance (or bound state) of the sugar–phosphate group leads to a strand break.

This, at first glance, surprising result, i.e., the lack of a parent anion signal for dCMPH where PT occurs, according to the tested hypothesis, an obstacle for electron-induced dissociation, may be explained by specific conditions of the CEMB experiment. Namely, it is worth noticing that anion yields are measured in the single collision regime, which means UHV conditions and, in consequence, the excess energy coming from the electron attachment process cannot be swiftly dissipated in the gas phase. Our B3LYP results demonstrate that the AEA for dCMPH amounts to 7.5 kcal/mol and is close to the activation barrier accompanying the H_2_PO_4_^–^ anion release from dCMPH^–^ (ΔG* = 9.4 kcal/mol; see Figure 8A). Apparently, the excess energy associated with the electron attachment process makes dissociation of the C5′–O bond in dCMPH^–^ quicker than a possible proton transfer.

### 3.3 Photoelectron Spectroscopy

**Figure 5:**
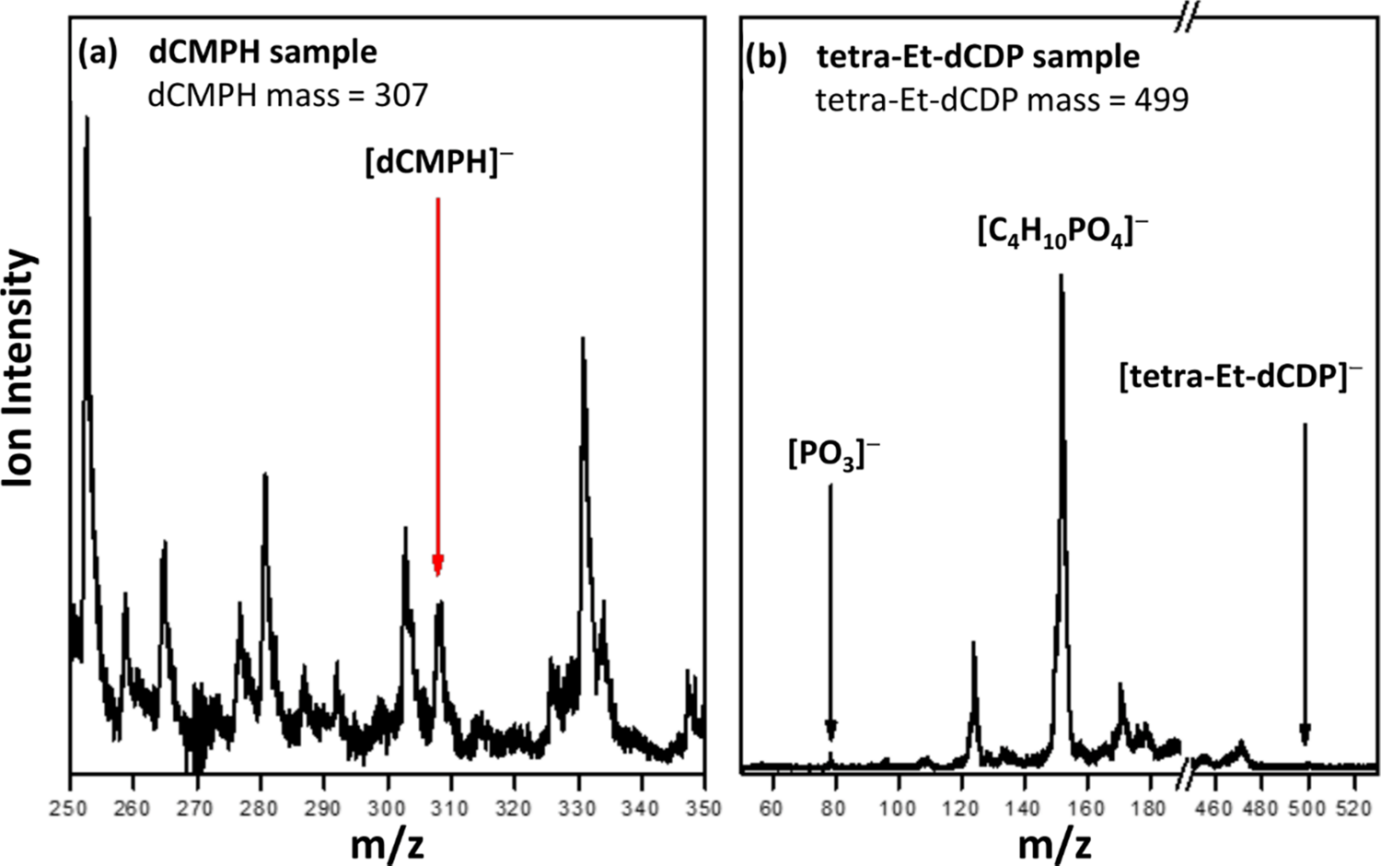
Mass spectra of (a) dCMPH and (b) tetra-Et-dCDP obtained by IR desorption and photoemission ion source.

In order to confirm the above-described reasoning and eventually deliver an experimental proof for EIPT in the studied systems, we decided to carry out PES experiments. Unlike the CEMB method, our aPES experiments utilized helium gas to provide a collisionally cooled jet to carry away excess energy and stabilize the resulting parent anions formed in the source. Hence, the energy related to the electron attachment process is quickly dissipated, which inhibits degradation paths opened under the CEMB conditions. Such an electron attachment process should ultimately lead to a qualitative difference between aPES results for the ethylated and native nucleotides since rapid transfer of the excess energy should allow for PT to proceed before C5′–O bond dissociation. Figure 5 shows the anionic mass spectra of dCMPH and tetra-Et-dCDP using our pulsed infrared (IR) desorption photoemission ion source. The parent anion of dCMPH with a mass-to-charge ratio of 307 was observed in Figure 5a. Not surprisingly, we were unable to generate the parent anions of tetra-Et-dCDP. The presence of the C_4_H_10_PO_4_^–^ anion demonstrates that electron attachment promoted the fragmentation of the ethylated, tetra-Et-dCDP derivative.

**Figure 6:**
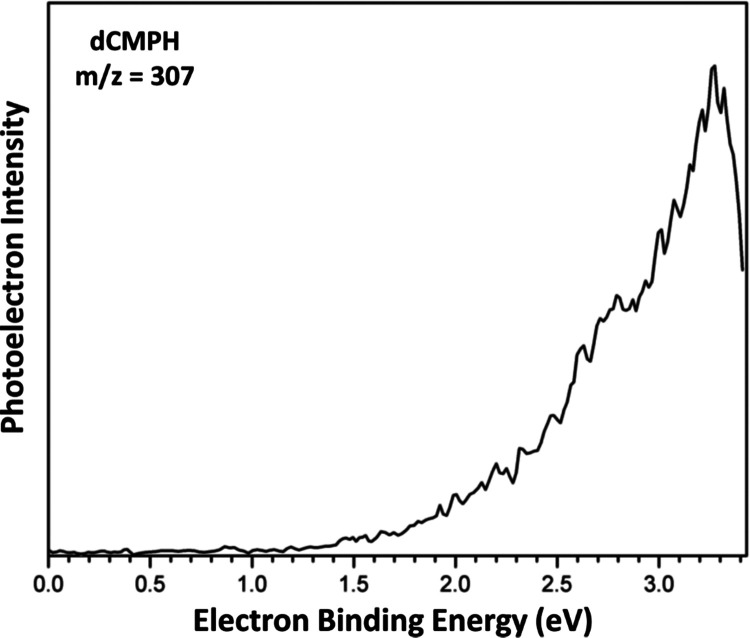
Photoelectron spectrum of dCMPH anions recorded with 3.49 eV photons.

The photoelectron spectrum of dCMPH recorded with 3.49 eV photons is shown in Figure 6. The spectrum exhibits an increasing band at the EBE scale starting from ∼1.5 eV with a maximum of 3.27 eV. A small shoulder occurs between ∼2.6 and 2.9 eV and peaks at ∼2.8 eV. The broad band results from the vertical photodetachment of the excess electron from the ground vibronic state of mass-selected anions to the ground vibronic state of the resulting neutrals. The maximal photoelectron intensities correspond to the optimal Franck–Condon overlaps of the vibrational wave functions between the anion and neutral ground states, leading to an energetic quantity known as the vertical detachment energy (VDE). Thus, the VDE value is determined to be 3.27 eV. The energy difference between the lowest vibrational level of the ground electronic state of the anion and the lowest vibrational level of the ground electronic state of its corresponding neutral is the AEA. However, the AEA value is hard to determine explicitly due to the absence of the resolved, assignable vibrational structure and the possible presence of vibrational hot bands in the spectrum. Nevertheless, as a reasonable approximation, one can estimate the AEA as that corresponding to the EBE value at ∼10% of the rising photoelectron intensity.^33^ Therefore, from the onset of the photoelectron spectrum, the AEA for dCMPH can be estimated to be ∼1.6 eV.

### 3.4 Computational Interpretation of CMEB and aPES Results

**Figure 7:**
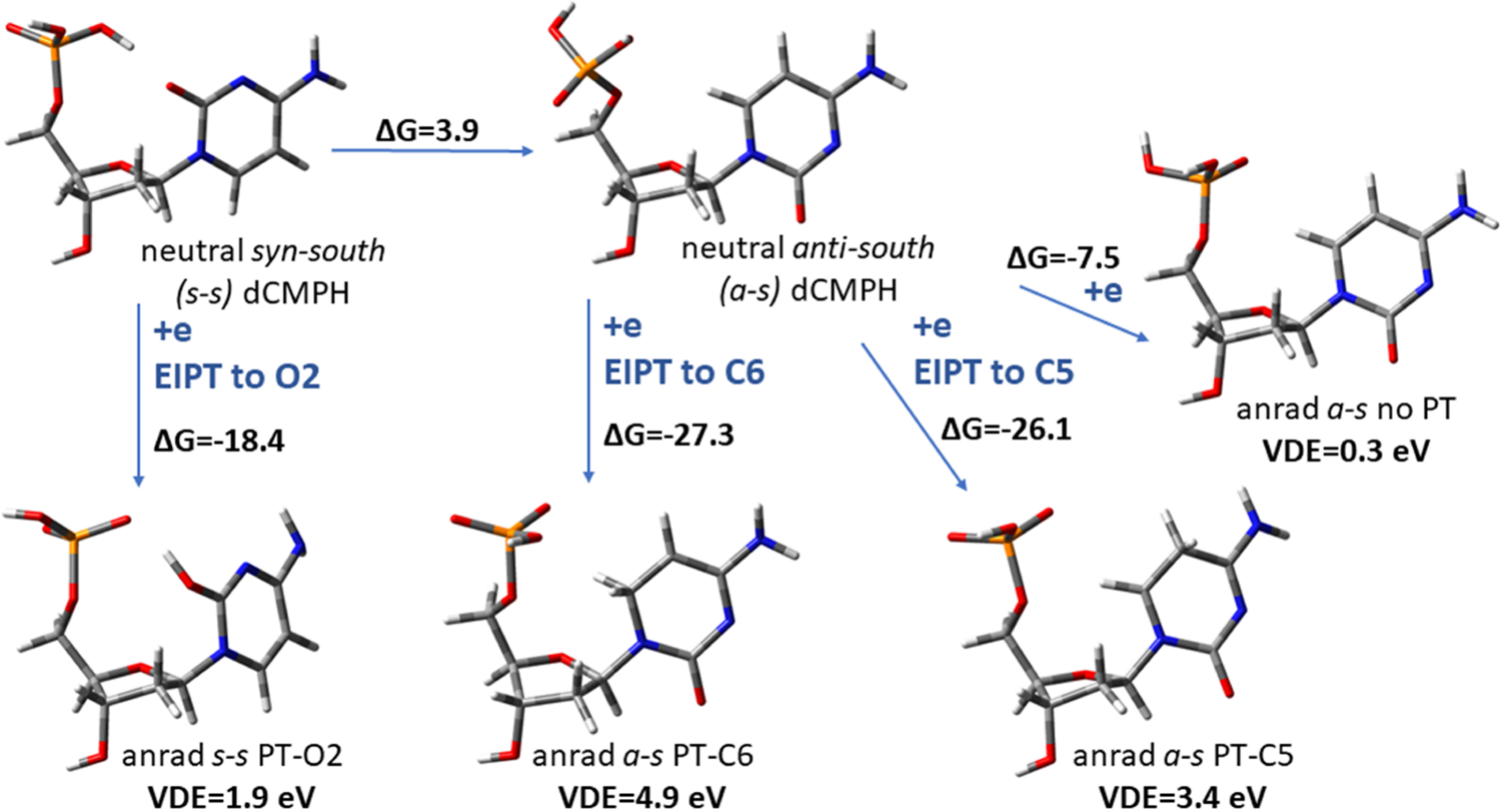
Visualization of the optimized neutral *syn*-*south* and *anti*-*south* conformers of 2′-deoxycytosine 5′-monophosphate (dCMPH), along with the possible paths for electron attachment-induced (EI) processes. Thermodynamic data are given in kcal/mol, while VDE is in eV. VDE values calculated at the B3LYP/6-31++G(d,p) level given below each anion radical system considered: the basic nonproton transferred anion (anrad *a*-*s* no-PT) and three anion radicals with the proton transferred from the phosphate group to the O2 site (anrad *s*-*s* PT-O2, barrier-free process), to the C6 site (anrad *a*-*s* PT-C6, barrier-free process), or to the C5 site (anrad *a*-*s* PT-C5).

The computational analysis of the electron attachment process to the dCMPH molecule shows that its anion radical is prone to transferring a proton from the phosphate moiety to the pyrimidine ring. We calculated VDE values for each of the obtained anion radicals, i.e., for those with proton transferred to the C5, C6, and O2 positions as well as for the non-PT structure (see Figure 7).

Our calculations suggest that the most stable anion radical isomer, anrad *a*-*s* PT-C6, could not be observed in the PES spectrum, as its VDE value is as high as 4.9 eV, which is too high to be detected with 3.49 eV photons used in the aPES experiment. The calculations indicate that the most intense peak, determined to be at 3.27 eV in the PES spectrum (see Figure 6), is related to the PT-C5 anion radical, which is only slightly less favorable than PT-C6 (Δ*G* ∼ 1 kcal/mol), and is characterized by 3.4 eV VDE value calculated at the B3LYP level (see Figure 7). The additional small shoulder above 2 eV, seen on the spectrum (Figure 6), could be due to the isomer for which the proton is transferred to the O2 atom (anrad *s*-*s* PT-O2; see Figure 7); however, its VDE (=1.9 eV) seems to be too small to explain the experimental feature at ca. 2.8 eV (Figure 6). In order to exclude methodological artifacts, we calculated VDE for anrad *s*-*s* PT-O2 at the CAM-B3LYP/aug-cc-pvtz, MN15/aug-cc-pvtz, and ωB97xD/aug-cc-pvtz levels and obtained its values equal to 1.88, 1.82, and 1.80 eV, respectively. One can therefore conclude that the VDE value obtained at B3LYP/6-31++G(d,p) is not an artifact related to the B3LYP functional. Since thermodynamic equilibrium is usually not attained in the aPES experiment, other low-energy structures may account for the measured signal. For instance, we calculated that the *a*-*s* PT-O2 isomer being ca. 5.5 kcal/mol less stable than the *s*-*s* PT-O2 one is characterized by the VDE of 2.3 eV, which is much closer to the experimental value. The basic structure of dCMPH (anrad *a*-*s* no-PT; see Figure 6) with its VDE of 0.3 eV is clearly not observed in the PES spectrum due to its lability and susceptibility to electron-induced barrier-free proton transfer, leading directly to the most stable anrad *a*-*s* PT-C6 isomer.

**Figure 8:**
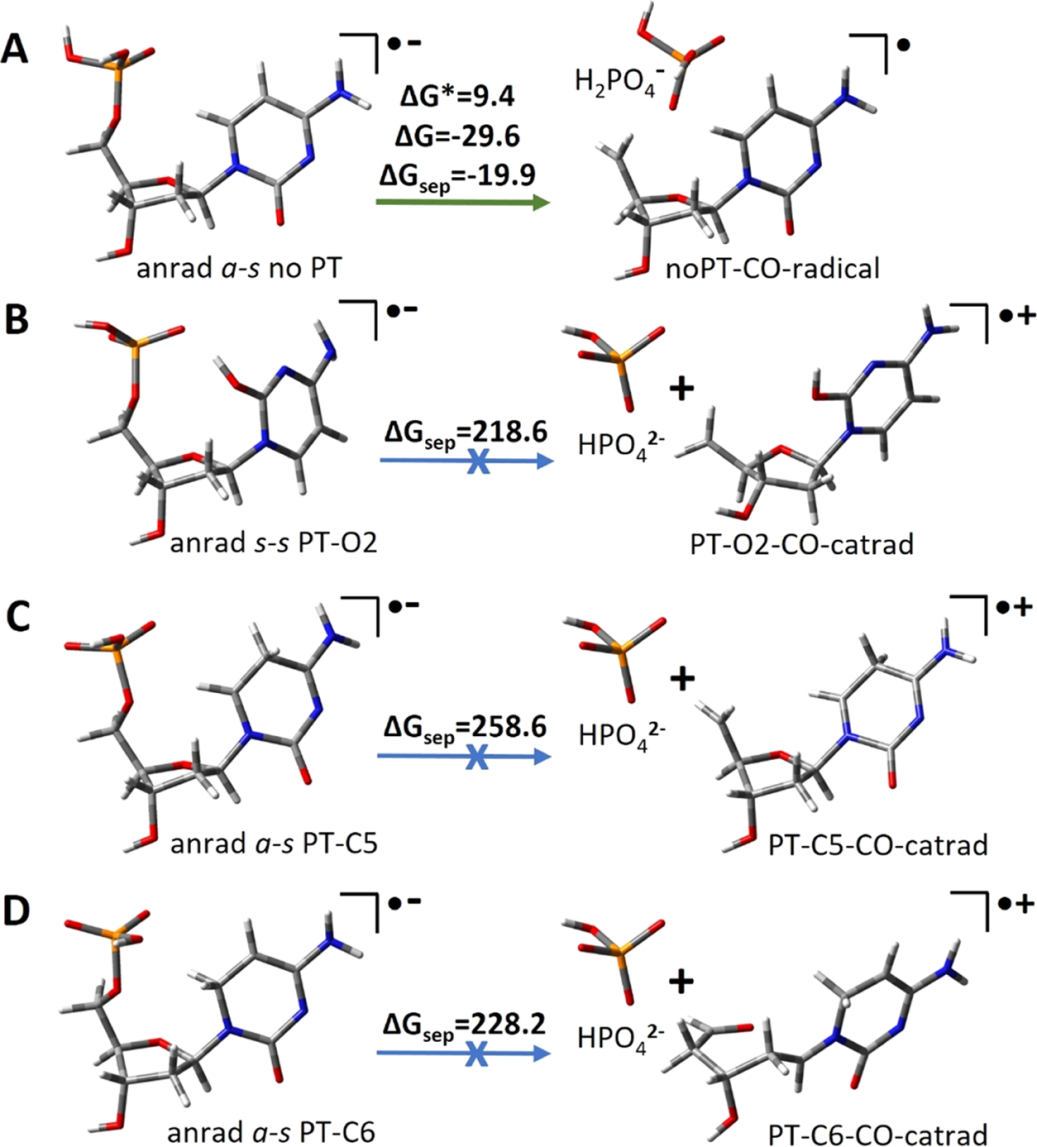
C5′–O bond breakage paths for the possible anion radical of dCMPH isomers. All thermodynamic and kinetic barriers are given in kcal/mol.

Electron attachment to the most stable neutral, *syn*-*south* conformer of dCMPH leads to barrier-free proton transfer from the phosphate group to the O2 atom of the pyrimidine ring, giving the anrad *s*-*s* PT-O2 anion radical (see Figure 7). On the other hand, electron attachment to the *anti*-*south* neutral conformer of dCMPH, which is 3.9 kcal/mol less stable (Gibbs free energy scale), also leads to barrier-free proton transfer from the phosphate group to the C6 atom of the pyrimidine ring, giving the most thermodynamically stable anion radical (see anrad *a*-*s* PT-C6, Figure 7). There is one more possible proton transfer process in the anion radical of dCMPH, competitive to PT to C6, i.e., PT to the C5 atom of the pyrimidine ring, geometrically possible also for the *anti*-*south* nucleotide conformation (see anrad *a*-*s* PT-C5 at Figure 7). Anrad *a*-*s* PT-C5 is only 1.2 kcal/mol (in the Gibbs free energy scale) less stable than anrad *a*-*s* PT-C6. Hence, together with the least stable anrad *a*-*s* no-PT isomer, we ended up with four anion radicals, which could potentially undergo further degradation via the C5′–O break (see Figure 8). It turned out that only the least stable no-PT isomer is prone to the sugar–phosphate bond cleavage (see path A in Figure 8). Breaking the C5′–O bond in the anrad *a*-*s* no-PT dCMPH isomer is both thermodynamically favorable (Δ*G* = −29.6 kcal/mol) and kinetically possible (activation barrier for this process is estimated to be only 9.4 kcal/mol; see panel A in Figure 8).

However, sugar–phosphate bond breakage becomes much less likely in the dCMPH anion radical isomers, where the proton is transferred to the pyrimidine ring (see panels B–D, Figure 8). Due to PT, the negative charge localized to the pyrimidine ring in the dCMPH parent anion is moved to the phosphate group. Under such circumstances, breaking the C5′–O bond would lead to the HPO_4_^2–^ moiety (and the corresponding cation radical residue; see Figure 8B–D) instead of H_2_PO_4_^–^ (and the corresponding radical; see Figure 8A), which was found to be much less stable in the gas phase, namely, the attempts of obtaining the thermodynamically stable complexes of proton-transferred dCMPH anion radicals, with the C5′–O bond breaking failed, as C5′–O reconnects during geometry optimization. To estimate the thermodynamic stimuli for the C5′–O breakage in PT structures, we calculated the Δ*G*_sep_ values, based on the separated product energetics, which was found to be as high as above 200 kcal/mol (Figure 8B–D). It clearly shows that the electron-induced internucleotide PT process prevents the SSB-type cleavage.

**Figure 9:**
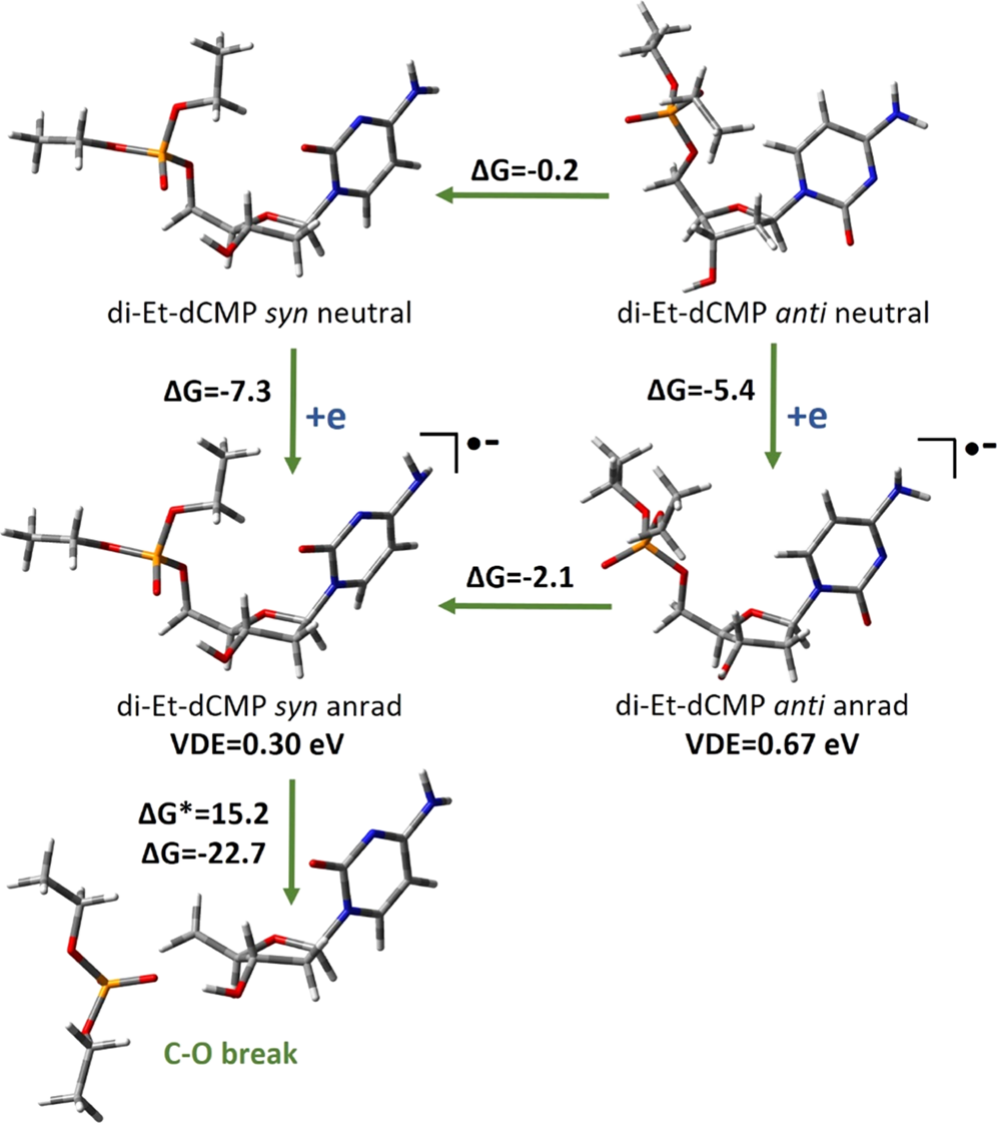
Electron attachment-induced degradation paths of di-Et-dCMP, along with thermodynamic (Δ*G*) and kinetic (ΔG*) data. All values (except VDE) are given in kcal/mol.

Different results are calculated in the cases of di-Et-dCMP and tetra-Et-dCDP cytidine derivatives, where proton donor hydroxyl groups at the phosphate moieties are ethylated (see Scheme 1). If PT is blocked that way, electron attachment to di-Et-dCMP leads to an electronically stable (AEA_G_ = 7.3 kcal/mol, VDE = 0.30 eV for the favorable *south*-*syn* conformer; see Figure 9) anion radical. Then, the di-Et-dCMP anion radical can easily undergo electron-induced degradation via the C5′–O bond break. Such degradation is both kinetically and thermodynamically favorable, with a reasonably low kinetic barrier (ΔG* = 15.2 kcal/mol) and high negative thermodynamical stimulus (Δ*G* = −22.7 kcal/mol; see Figure 9).

**Figure 10:**
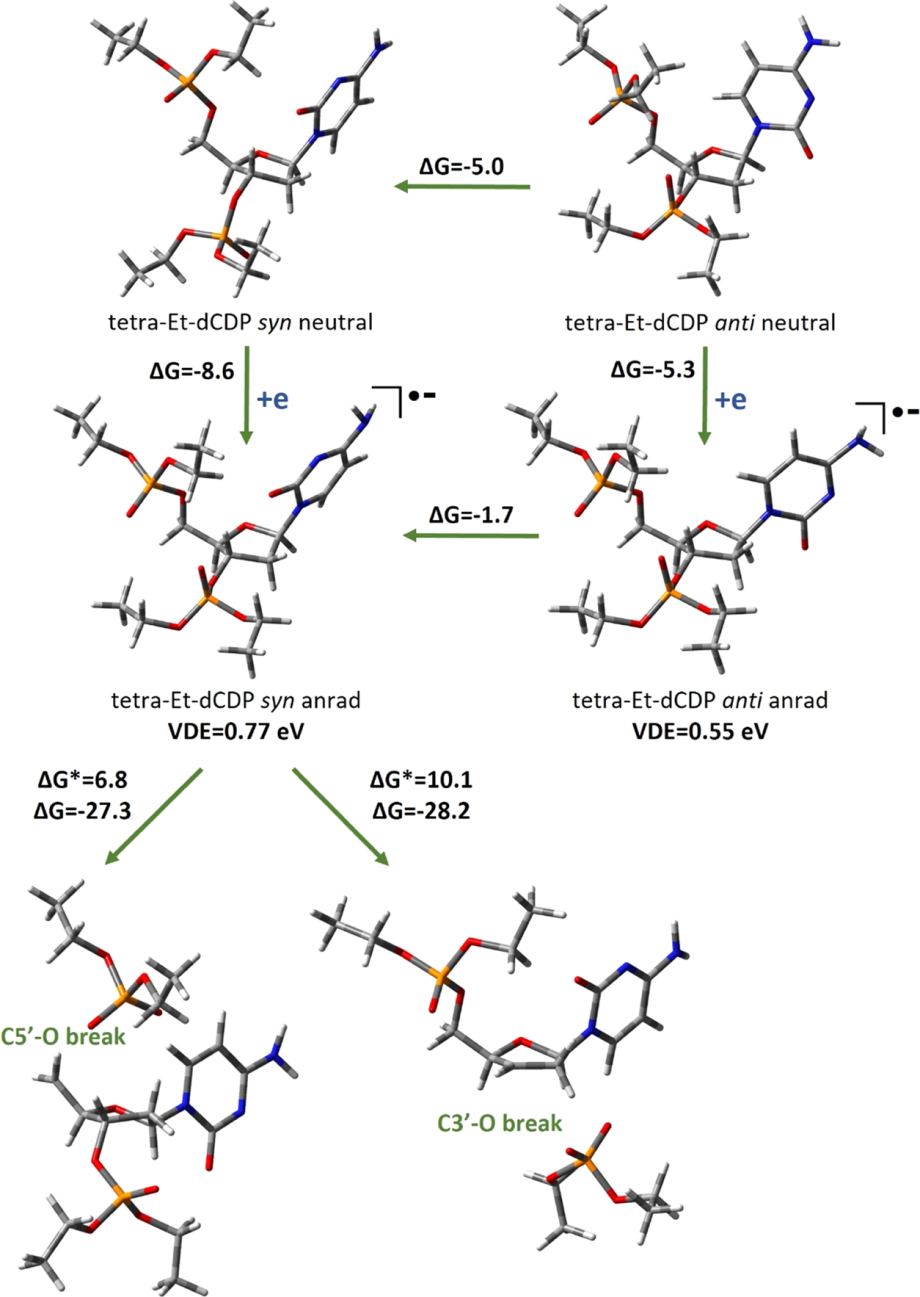
Electron attachment-induced favorable C–O break-type degradation paths of tetra-Et-dCDP, along with thermodynamic (Δ*G*) and kinetic (ΔG*) data. All values (except VDE) are given in kcal/mol.

Electron attachment-induced degradation looks similar in the case of the tetra-Et-dCDP compound. Once again, more thermodynamically stable are the *south*-*syn* conformers, in both neutral and anionic radical forms, and the tetra-Et-dCDP electron affinity is positive (AEA_G_ = 8.6 kcal/mol, VDE = 0.77 eV; see Figure 10). The tetra-Et-dCDP anion radical formed after electron attachment can dissociate via the C5′–O or C3′–O bond breakage, which are equivalents of SSB in DNA. Both degradation paths are thermodynamically allowed, with high negative thermodynamic stimuli (Δ*G* = −27.3 vs Δ*G* = −28.2 kcal/mol for, respectively, C5′–O and C3′–O bond breaks). Both activation barriers are low, and a slightly lower activation barrier for the C5′–O break (ΔG* = 6.8 vs ΔG* = 10.1 kcal/mol, see Figure 10) suggests that the C5′ site of the nucleoside is more prone to electron-induced SSB, but only if the proton transfer process to a nucleobase is hindered.

## 4 Conclusions

The current study was undertaken to confirm the hypothesis according to which EIPT processes block the dissociation of the C*X*–O bond, which mimics a single-strand break formation in DNA. This hypothesis, if valid, would explain the experimentally confirmed fact that electron attachment to dry DNA may produce SSBs, while an identical reaction does not occur in an aqueous solution containing the biopolymer.

In order to support the above-mentioned proposition, we have studied three molecular systems, the native dCMPH nucleotide as well as the ethyl esters of mono- and diphosphate of 2′-deoxycytidine, di-Et-dCMP, and tetra-Et-dCDP, with CEMB and aPES experiments, involving the electron attachment process as well as with the DFT modeling. In dCMPH, proton transfer induced by electron attachment is feasible, while such a possibility is blocked in the ethylated derivatives. In any case, the CEMB results do not differentiate between the native nucleotide and respective esters. In the three studied systems, the most intensive degradation process is related to the C*X*–O bond cleavage in the respective anions. Moreover, a parent anion is not observed, while it should be present under CEMB conditions if the DEA process is blocked by EIPT. However, a single collision regime employed in the CEMB experiments prevents dissipation of the excess energy related to electron attachment, which is probably responsible for the observed instability of the dCMPH anion. Hence, the CEMB measurements do not allow for the full confirmation of the tested hypothesis.

Quite different behavior is observed in the aPES experiments. Here, the parent anions were not detected for the ethylated derivatives, where PT is inhibited. In line with the CEMB experiments, only the ion signal related to the release of the ethylated phosphate anions is observed instead. For dCMPH, however, the ion signal for its parent anion was detected and its PES spectrum was measured. The calculated VDEs suggest that the PES peaks at 2.8 and 3.27 eV come from the proton-transferred structures. Altogether, these results support the EIPT hypothesis.

In summary, for the first time, we demonstrated that electron attachment-induced proton transfer changes the propensity of an anionic nucleotide to undergo C*X*–O bond cleavage, and by extrapolating this finding to the DNA molecule, one may suggest that SSBs are formed due to excess electron attachment only when the respective proton transfer cannot occur. This statement remains consistent with the experimentally observed differing sensitivities of dry and wet DNA to excess electrons.

In order to make our models more reliable (we studied an intramolecular PT so far), analogous measurements and calculations should be carried out for water clusters of nucleotides. Indeed, in an aqueous solution, the postulated PT may proceed between water molecules and the nucleotide anion (or the DNA electron adduct) rather than intramolecularly. For EIPT to occur, a sufficiently large water cluster must be considered in which the hydration energy of the hydroxyl anion, forming as a product of the PT process, would compensate for the energy of the proton transfer reaction.

## Supporting Information Available

^1^H, ^31^P, and ^13^C NMR spectra, MS spectra, HPLC chromatograms, and UV spectra of synthesized compounds; anion efficiency curves upon electron attachment to the ester of dCMP and dCDP; summary of fragment anions in terms of mass, structural assignments, peak maxima, and thermodynamic thresholds for the ester of dCDP; molecular and structural equations of possible anion formation pathways, and calculated positions of the shape resonances (PDF)
