# Supplementary material for: Intramolecular Proton Transfer in the Radical Anion of Cytidine Monophosphate Sheds Light on the Sensitivities of Dry vs Wet DNA to Electron Attachment-Induced Damage
Source: J Am Chem Soc. 2023 Apr 11;145(16):9059–71. doi: 10.1021/jacs.3c00591 (PMC10141262; doi:10.1021/jacs.3c00591)

Supporting Information

Intramolecular Proton Transfer in the Radical Anion of Cytidine Monophosphate Sheds Light on the Sensitivities of Dry versus Wet DNA to Electron Attachment-Induced Damage

Lidia Chomicz-Mańka,[1] Anna Czaja,[1] Karina Falkiewicz,[1] Magdalena Zdrowowicz,[1] Karol Biernacki,[2] Sebastian Demkowicz,[2*] Farhad Izadi,[3] Eugene Arthur-Baidoo,[3] Stephan Denifl,[3*] Zhaoguo Zhu,[4] Burak Ahmet Tufekci,[4] Rachel Harris,[4] Kit H. Bowen[4*], Janusz Rak[1*]

[1]Laboratory of Biological Sensitizers, Department of Physical Chemistry, Faculty of Chemistry, University of Gdańsk, Wita Stwosza 63, 80-308 Gdańsk, Poland

[2]Department of Organic Chemistry, Faculty of Chemistry, Gdańsk University of Technology, Narutowicza 11/12, 80-233 Gdańsk, Poland

[3]Institut für Ionenphysik und Angewandte Physik and Center for Biomolecular Sciences Innsbruck, Leopold-Franzens Universität Innsbruck, Technikerstrasse 25, A-6020 Innsbruck, Austria

[4]Department of Chemistry, Johns Hopkins University, Baltimore, Maryland 21218, United States.

[*]To whom correspondence should be addressed, e-mail: janusz.rak@ug.edu.pl

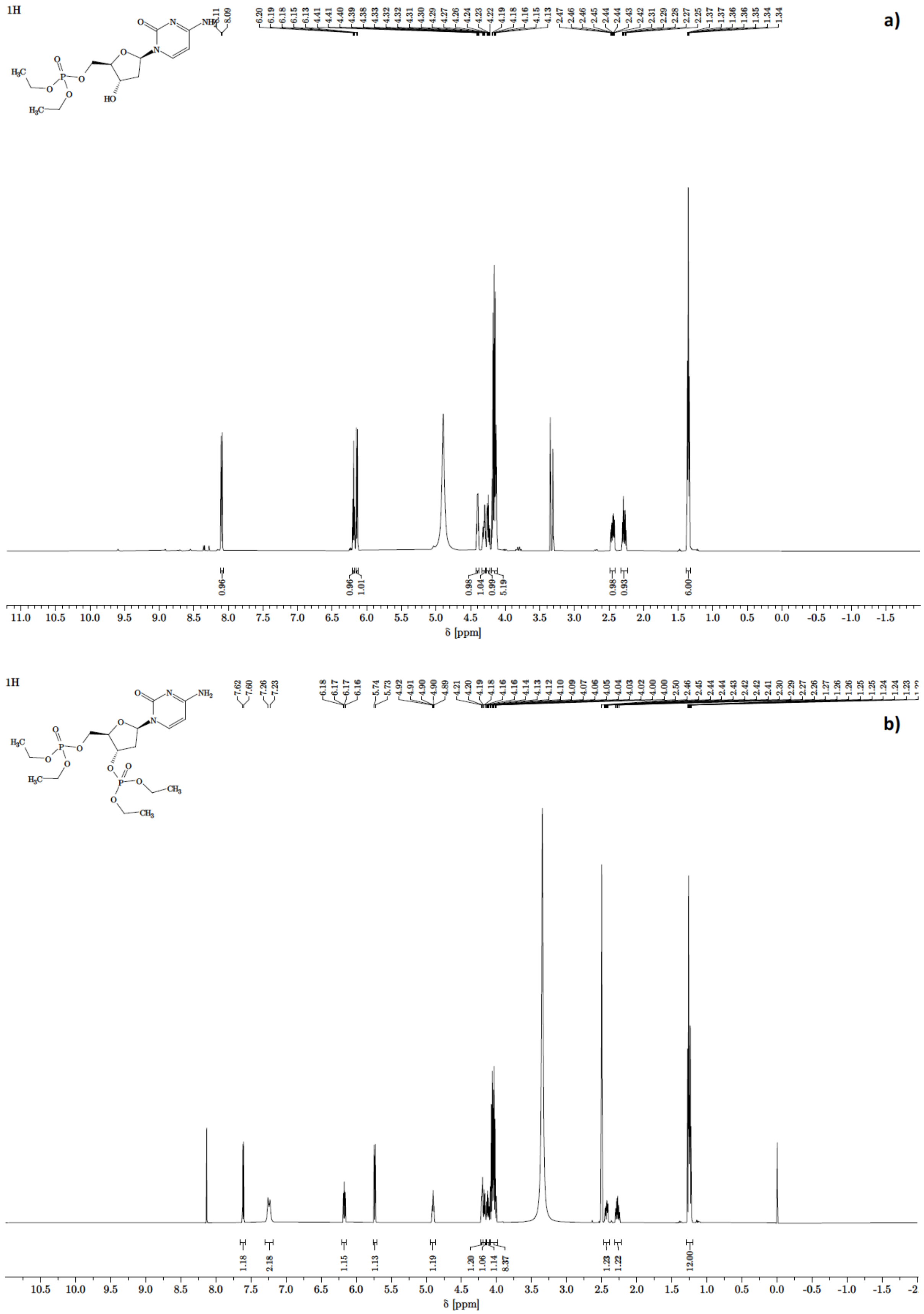

Figure S1. ^{1}H NMR spectra of a) di-Et-dCMP and b) tetra-Et-dCDP.

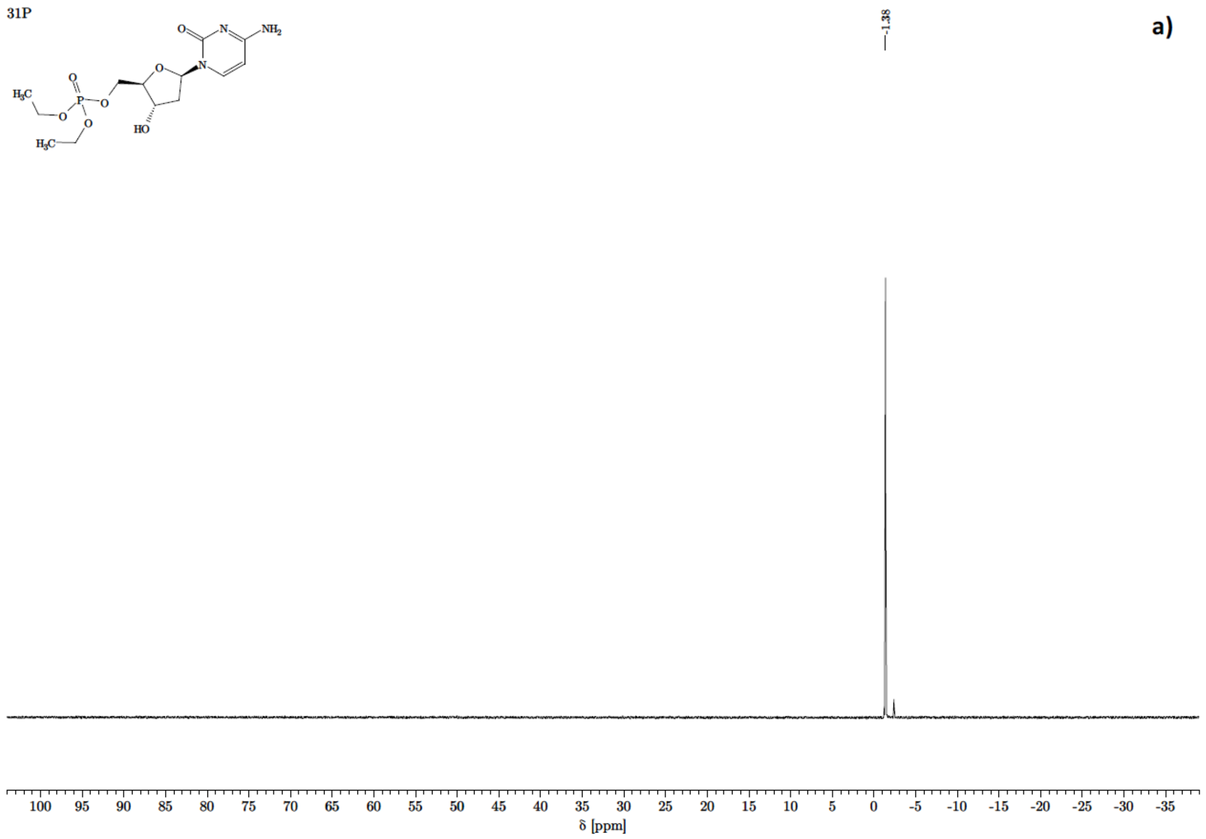

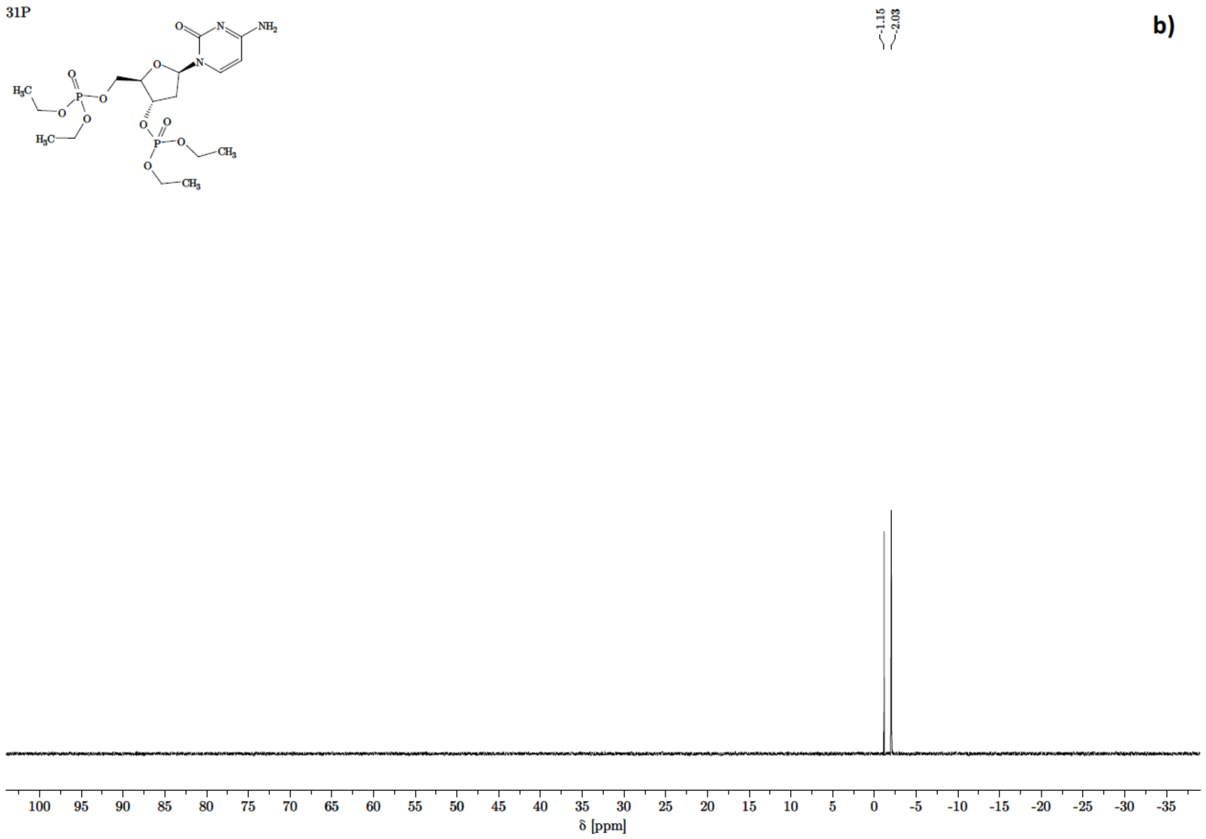

Figure S2. ^{31}P NMR spectra of a) di-Et-dCMP and b) tetra-Et-dCDP.

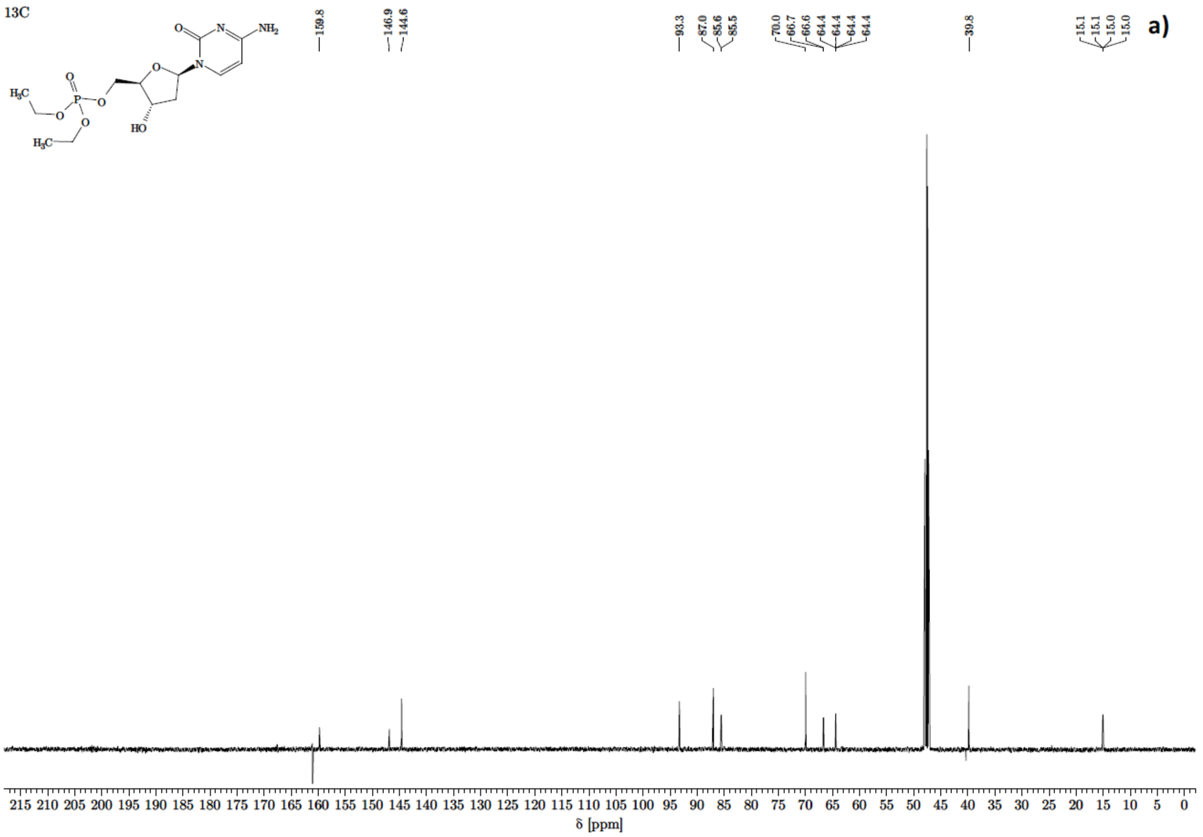

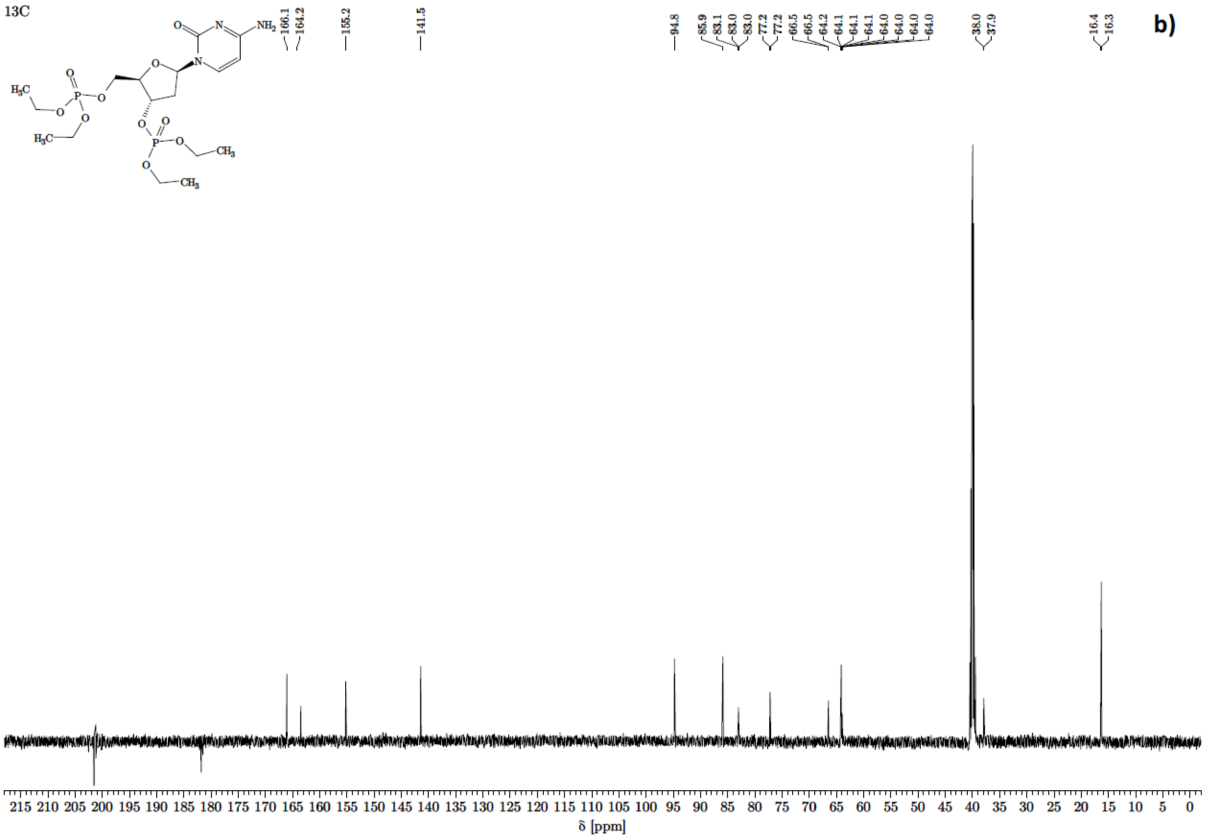

Figure S3. ^{13}C NMR spectra of a) di-Et-dCMP and b) tetra-Et-dCDP.

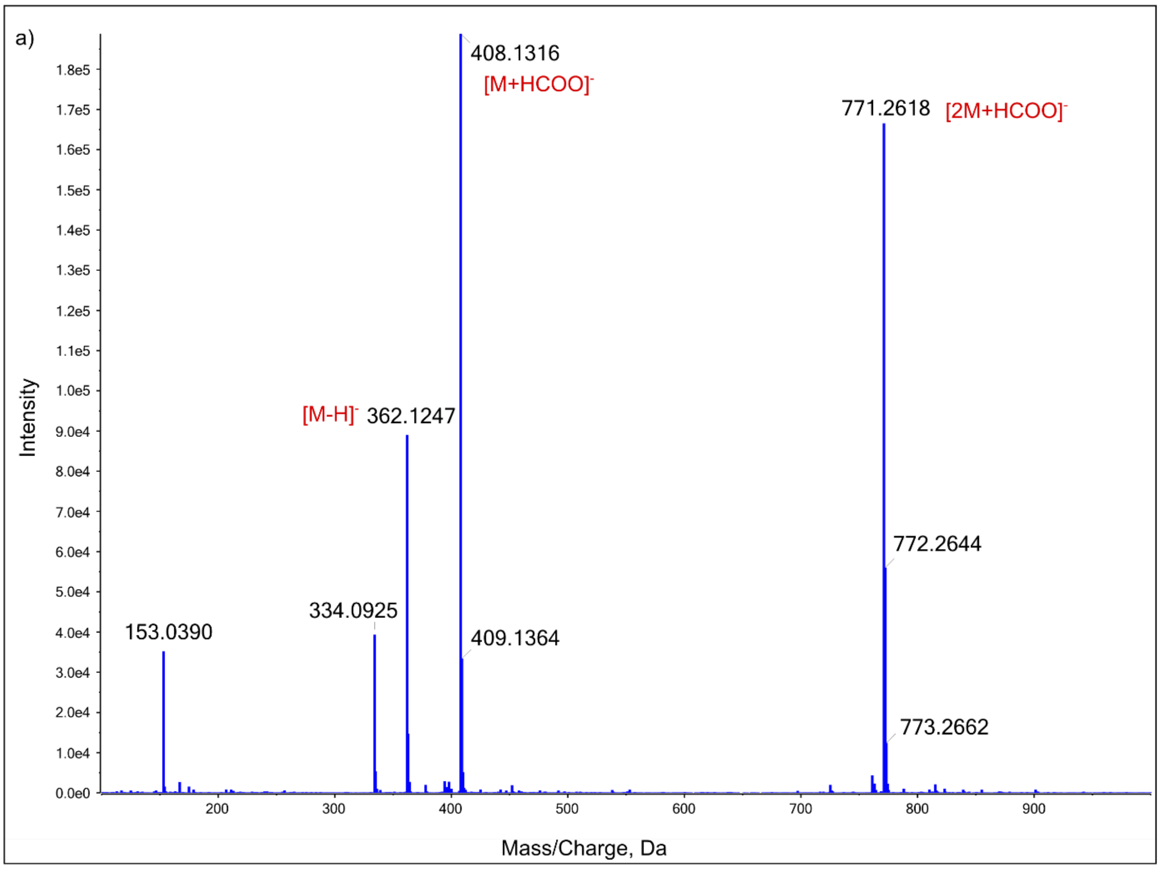

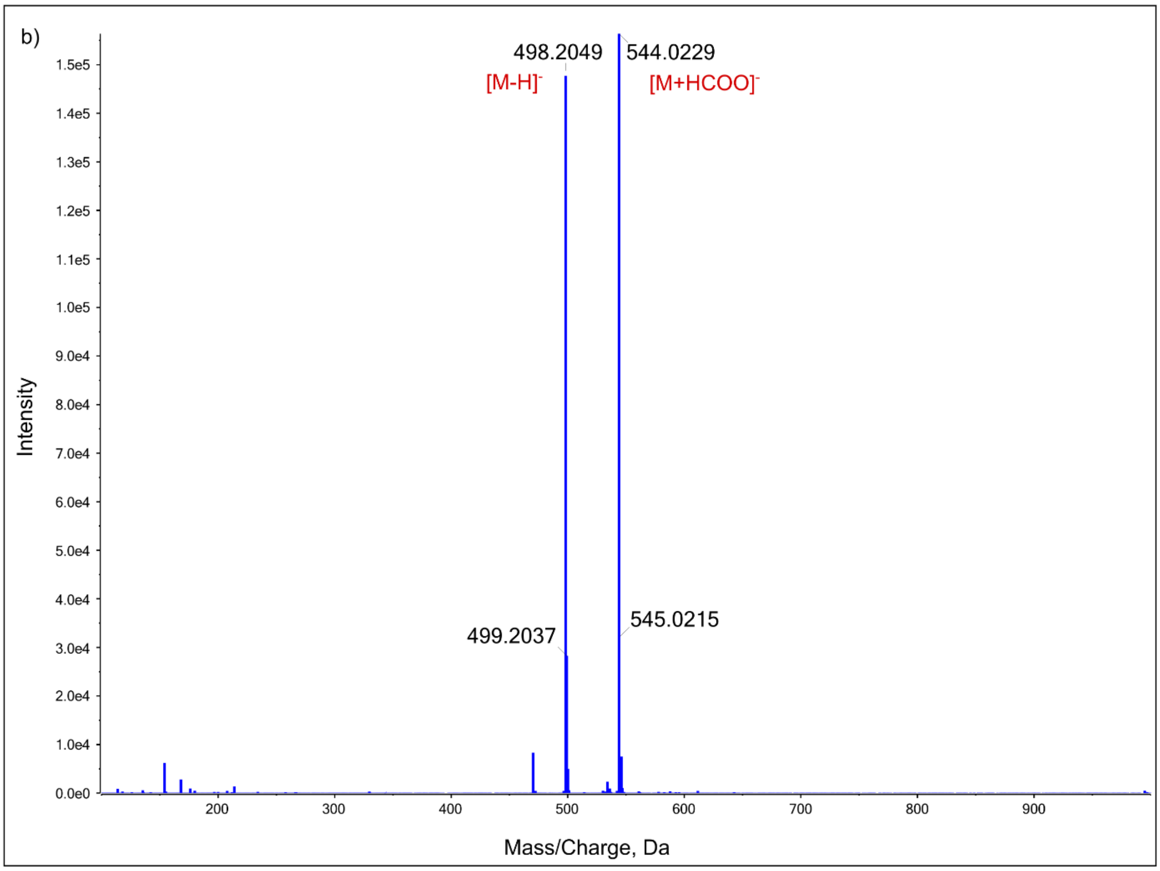

Figure S4. MS spectra of a) di-Et-dCMP and b) tetra-Et-dCDP.

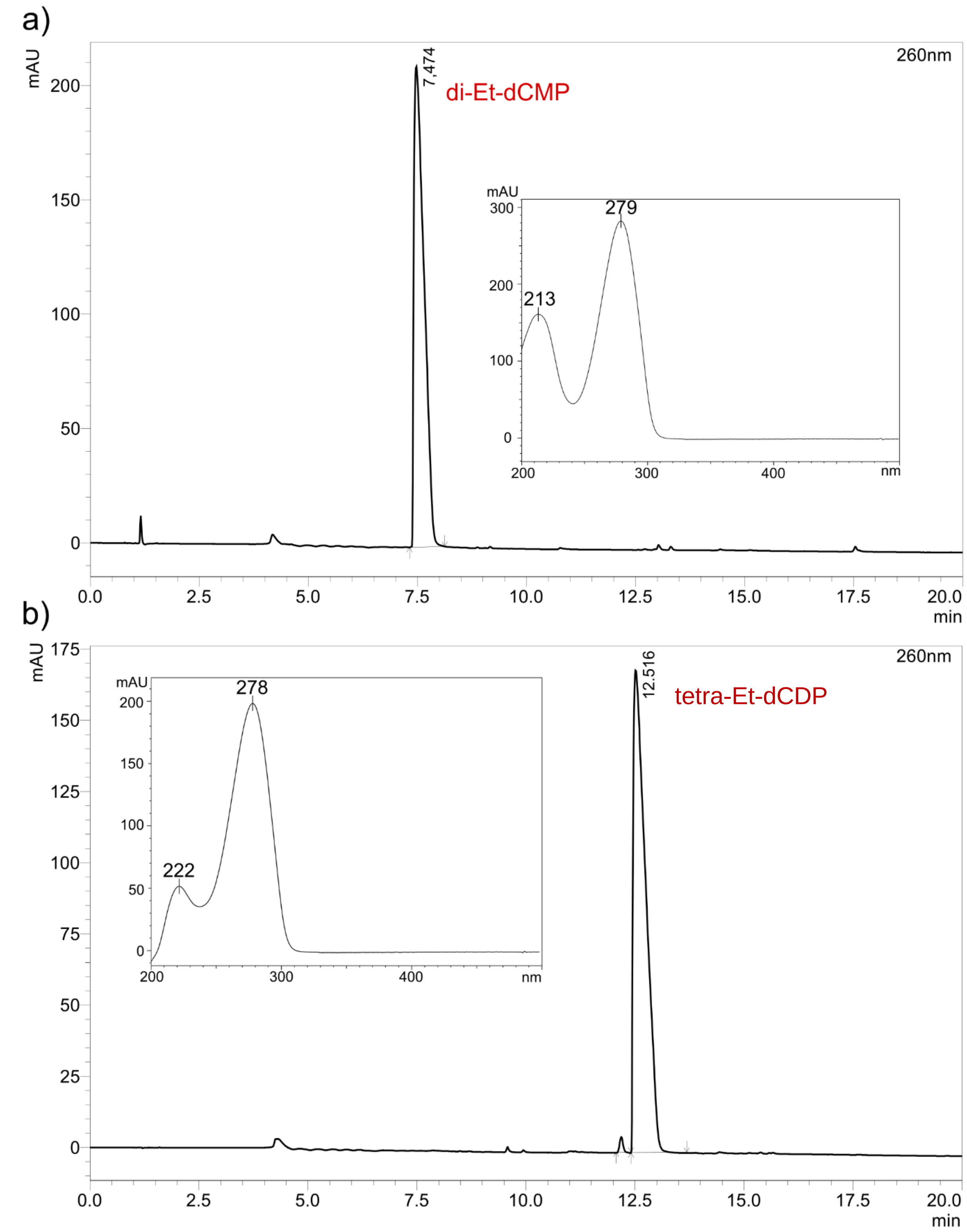

Figure S5. UHPLC chromatograms and UV-vis spectra of a) di-Et-dCMP and b) tetra-Et-dCDP.

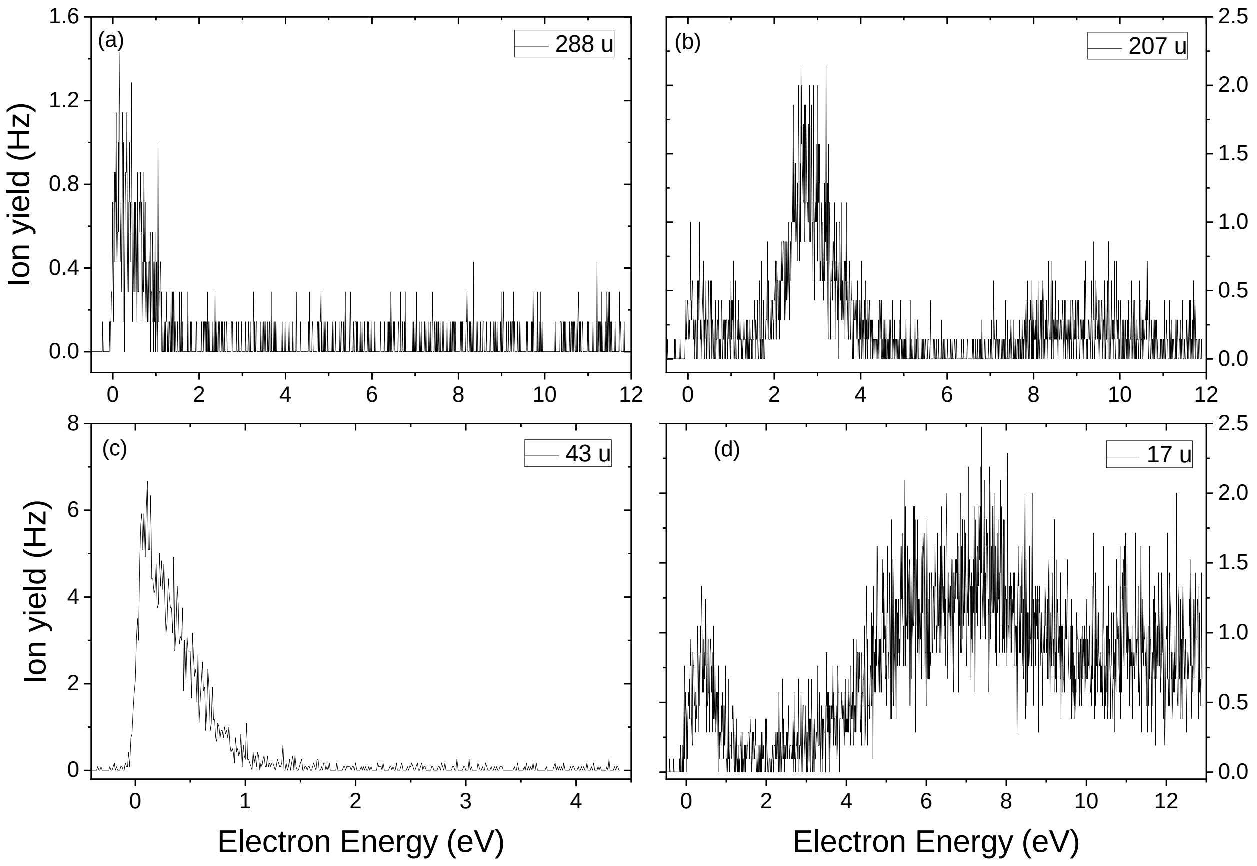

Figure S6. Anion efficiency curves for the formation of the anion with masses (a) 288 u, (b) 207 u, (c) 43u, and (d) 17 u upon electron attachment to the ester of dCMP (di-Et-dCMP).

Table S1. Summary of fragment anions in terms of mass, structural assignments, peak maxima and thermodynamic thresholds for the ester of dCMP (di-Et-dCMP). The main dissociation channel is marked with red color and is discussed in the main text. For the other fragment anions, which are much less abundant than the main product, a significant deviation of the experimental threshold and the calculated reaction enthalpy is notable. This deviation can be explained by two possible reasons, (i) the presence of impurities in the experiment, which lead to the formation of these anions or (ii) other reaction pathways in DEA to di-Et-dCMP lead to the formation of the ion yield at a certain m/z value than the calculated ones.

Mass m/z	Anion of di-Et-dCMP	Maxima of peak positions (eV)						Enthalpy (ΔH) [eV]		
		1.	2.	3.	4.	5.	6.	Exp. 360.95 K	Calc. 298.15 K	Calc. 360.95 K
288	$(C_9H_{11}N_3O_6P)^-$	0.15	0.59	--	--	--	--	~0	0.66	0.66
207[b]	$(C_8H_{16}O_4P)^-$	0.38	2.72	8.29	9.42		--	~0	-	-
**153**	**$(C_4H_{10}O_4P)^-$**	**0.05**	**1.21**	**2.52**				**~0**	**0.21**	**0.21**
45	$(C_2H_5O)^-$	1.32	1.64	2.17				1.1[a]	3.24	3.23
43	$(C_2H_3O)^-$	0.07	0.22	0.44				~0	3.79	3.79
17	$(OH)^-$	0.43	5.32	7.33	11.69	--	--	~0	2.70	2.71

[a] Different experimental parameters: 45 m/z (T = 315.15 K).
[b] 207 m/z: Anion is unstable, it degrades during the optimization.

Molecular and structural equations of possible anion formation pathways (charge and multiplicity marked blue):

$$\mathrm{di-Et-dCMP + e^- \rightarrow (di-Et-dCMP)^{*-} \rightarrow (C_9H_{11}N_3O_6P)^- + C_2H_5^{\bullet} + C_2H_5OH} \qquad (288\ m/z)$$

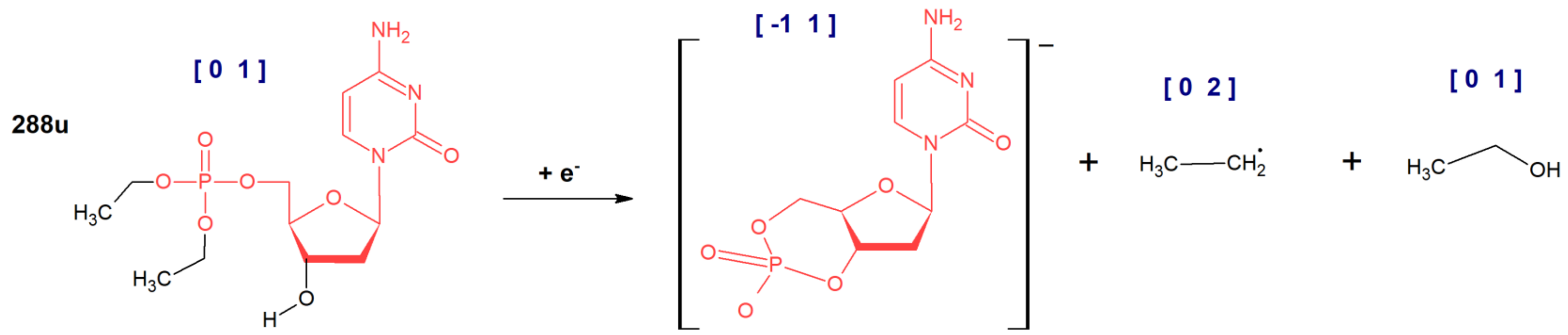

$$\mathrm{di-Et-dCMP + e^- \rightarrow (di-Et-dCMP)^{*-} \rightarrow (C_4H_{10}O_4P)^- + (C_9H_{12}N_3O_3)^{\bullet}} \qquad (153\ m/z)$$

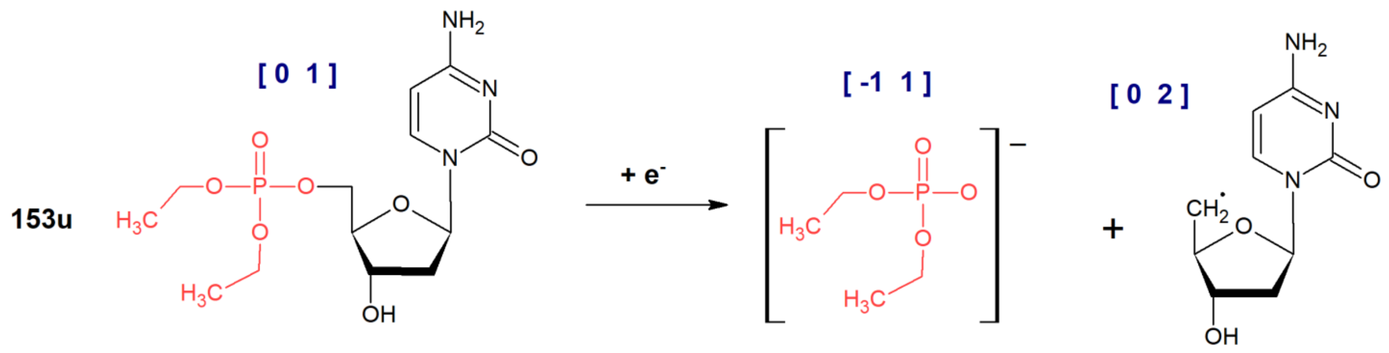

$$\mathrm{di-Et-dCMP} + e^{-} \rightarrow (\mathrm{di-Et-dCMP})^{*-} \rightarrow (C_2H_5O)^{-} + (\mathrm{di-Et-dCMP} - C_2H_5O)^{\bullet} \quad (45\ m/z)$$

45u

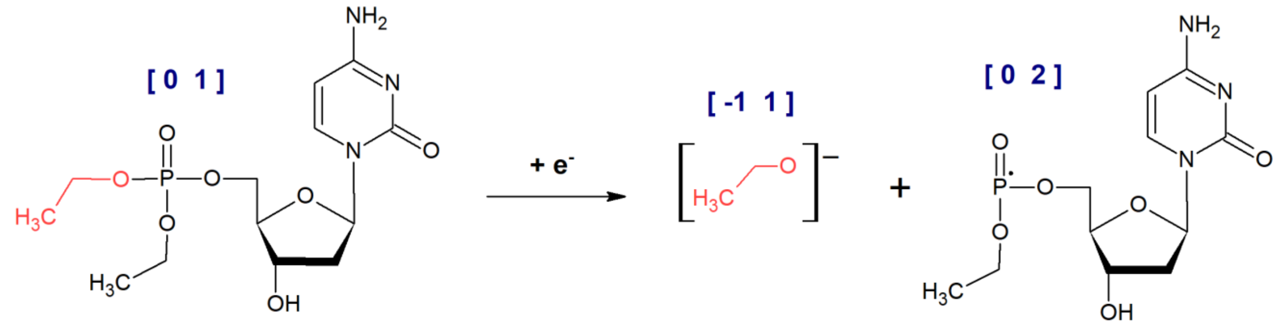

$$\mathrm{di-Et-dCMP} + e^{-} \rightarrow (\mathrm{di-Et-dCMP})^{*-} \rightarrow (C_2H_3O)^{-} + (C_4H_{10}O_4P)^{\bullet} + C_7H_9N_3O_2 \quad (43\ m/z)$$

43u

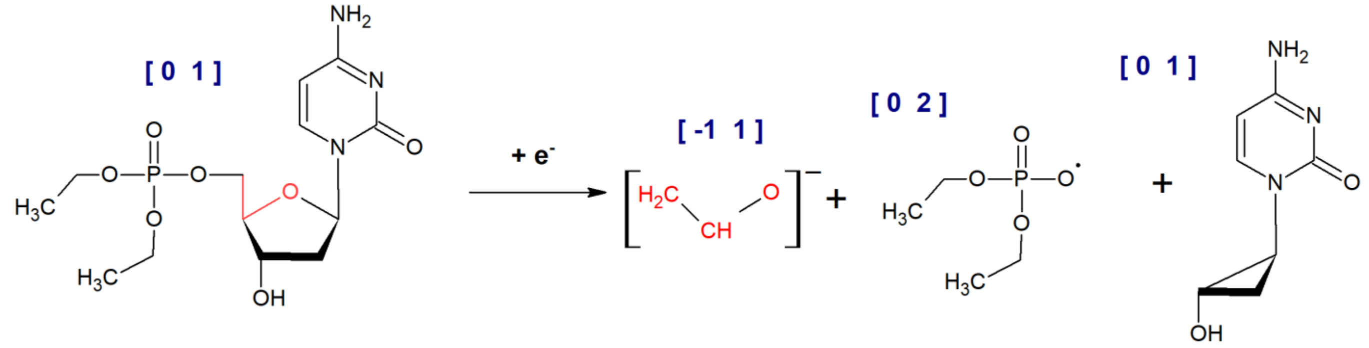

$$di - Et - dCMP + e^- \rightarrow (di - Et - dCMP)^{*-} \rightarrow (OH)^- + (di - Et - dCMP - OH)^{\bullet} \qquad (17\ m/z)$$

17u

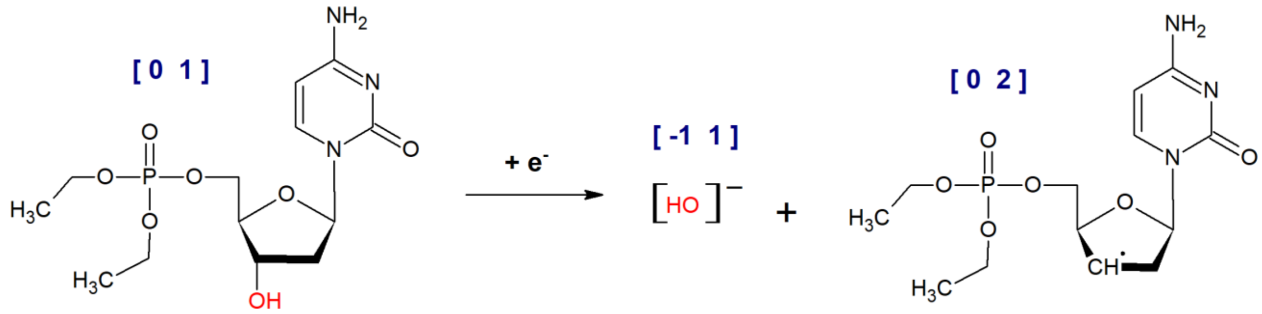

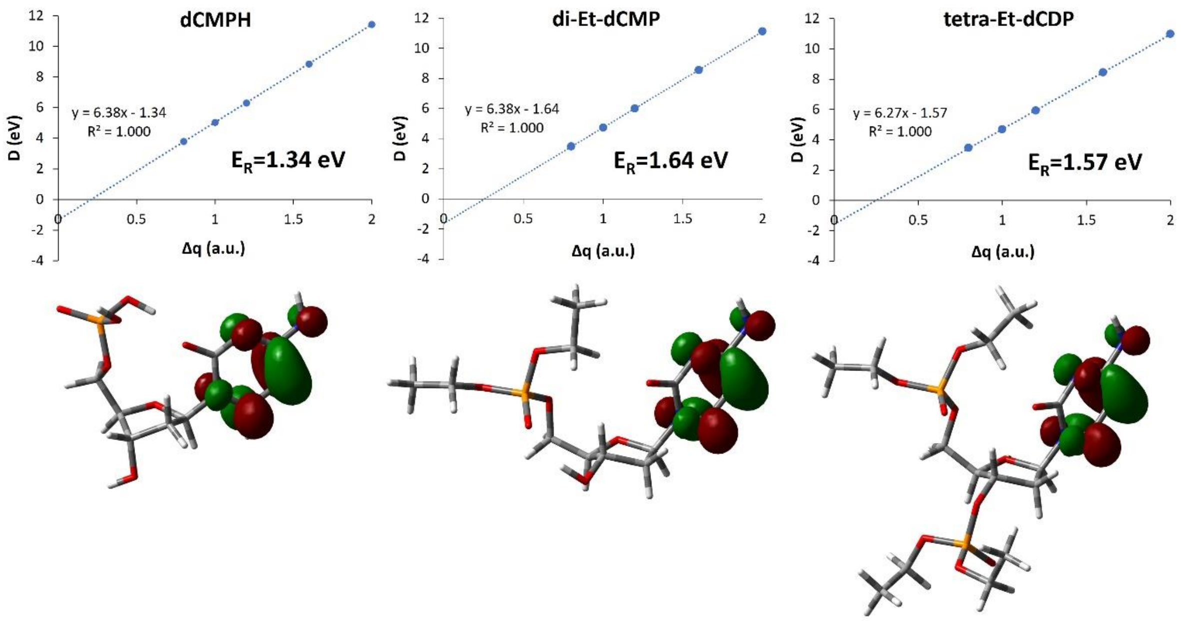

Figure S7. Binding energy, D, plotted against the stabilizing charge, Δq, values for which the anion is electronically stable and extrapolated to $\Delta q \rightarrow 0$. SOMO orbital visualized with the contour value of 0.05 $(a_0)^{-3/2}$ for Δq = 1.0. E_R stands for the calculated position of the resonance related to the release of the phosphorate group (97 m/z) in the case of dCMPH and the ethylated phosphorate group (153 m/z) in the case of di-Et-dCMP and tetra-Et-dCDP.

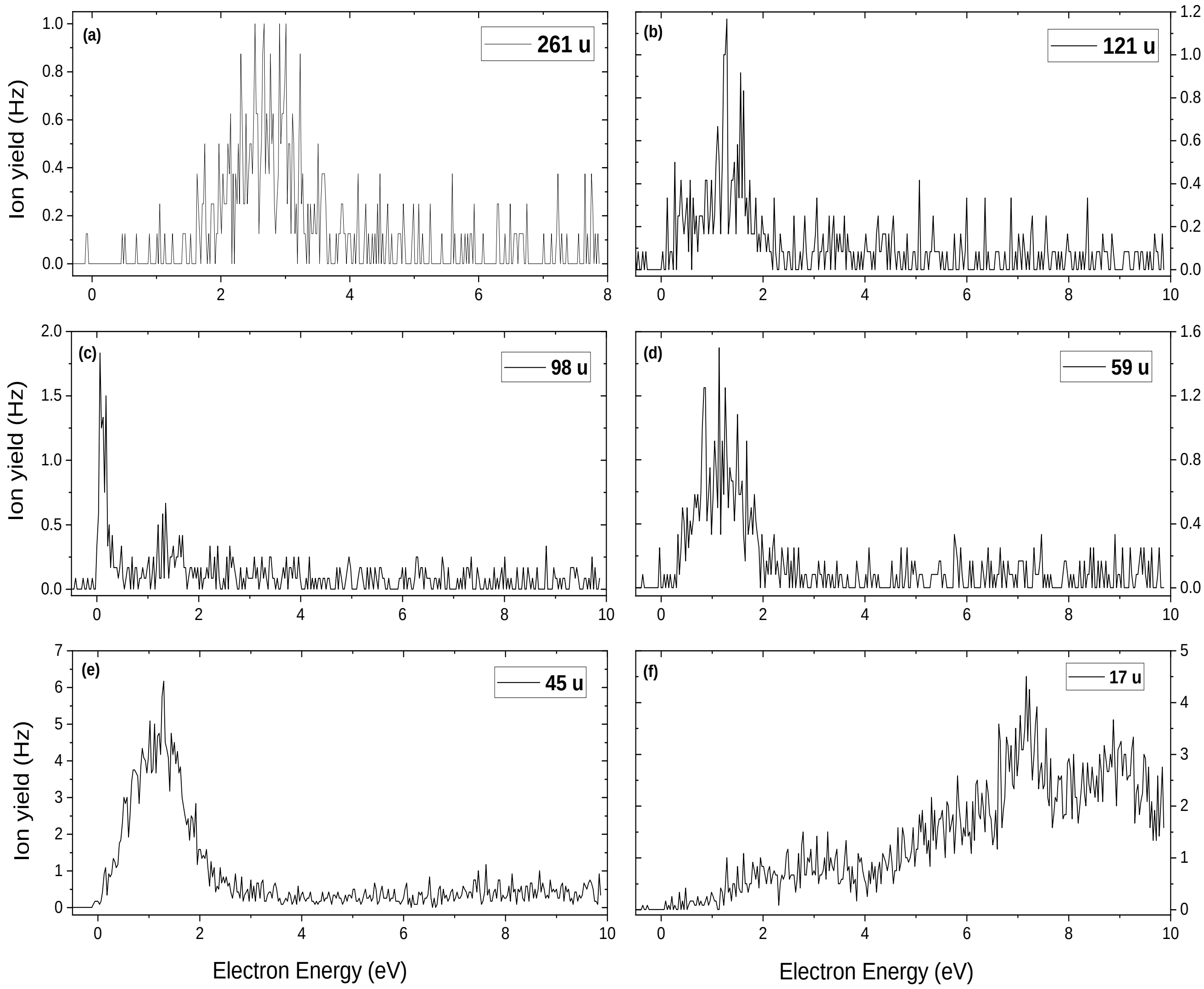

Figure S8. Anion efficiency curves for the formation of the anion with masses (a) 261 u, (b) 121 u, (c) 98 u, (d) 59 u, (e) 45 u and (f) 17 u upon electron attachment to the ester of dCDP (tetra-Et-dCDP).

Table S2. Summary of fragment anions in terms of mass, structural assignments, peak maxima and thermodynamic thresholds for the ester of dCDP (tetra-Et-dCDP). The main dissociation channel marked red. For the other fragment anions, which are much less abundant than the main product, a significant deviation of the experimental threshold and the calculated reaction enthalpy is notable. This deviation can be explained by two possible reasons, (i) the presence of impurities in the experiment, which lead to the formation of these anions or (ii) other reaction pathways in DEA to tetra-Et-dCMP lead to the formation of the ion yield at a certain m/z value than the calculated ones.

Mass m/z	Anion of tetra-Et-dCDP	Maxima of peak positions (eV)							Enthalpy (ΔH) [eV]	
								Exp.	Calc.	
		1.	2.	3.	4.	5.	6.	363.35 K	298.15 K	363.35 K
261	$(C_7H_8N_3O_6P)^-$	2.69	--	--	--	--	--	1.34	0.03	0.03
**153**	**$(C_4H_{10}O_4P)^-$**	**0.06**	**1.24**	**2.30**	**4.45**		**--**	**~0**	**-0.37 (1)[a]; -0.15 (2)**	**-0.37 (1); -0.15 (2)**
121	$(C_6H_5N_2O)^-$	0.42	1.31	4.53	--	--	--	0.06	3.84	3.85
98	$(C_5H_6O_2)^-$	0.11	1.43	--	--	--	--	~0	3.78	3.79
59	$(C_2H_3O_2)^-$	1.16		--	--	--	--	0.18	4.14	4.15
45	$(C_2H_5O)^-$	0.80	1.33	2.61	5.11	8.59		~0	2.81 (1)[b]; 2.83 (2)	2.81 (1): 2.83 (2)
17	$(OH)^-$	1.99	3.16	7.16	9.06		--	0.34	4.12 (1)[c]; 4.06 (2)	4.14 (1); 4.08 (2)

[a] threshold (1) for anion $(C_4H_{10}O_4P)^-$ release from C5' site; (2) from C3' site.
[b] threshold (1) for anion $(C_2H_5O)^-$ release from C5' site; (2) from C3' site.
[c] threshold (1) for anion OH^- release from C5' site (P=O and H from CH_3); (2) from C3' site.

Molecular and structural equations of possible anion formation pathways (charge and multiplicity marked blue):

$$\text{tetra}-\text{Et}-\text{dCDP}+e^{-}\rightarrow(\text{tetra}-\text{Et}-\text{dCDP})^{*-}\rightarrow(C_7H_8N_3O_6P)^{\bullet-}+C_{10}H_{23}O_4P \qquad (261\ \text{m/z})$$

261u

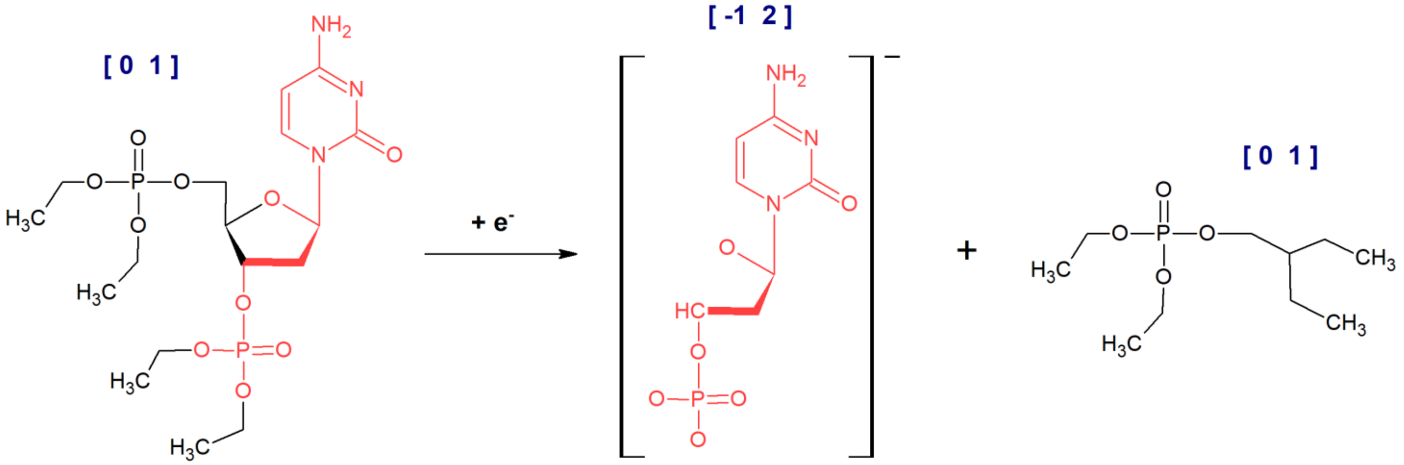

$$\text{tetra} - \text{Et} - \text{dCDP} + e^{-} \rightarrow (\text{tetra} - \text{Et} - \text{dCDP})^{*-} \rightarrow (C_4H_{10}O_4P)^{-} + (C_{13}H_{21}N_3O_6P)^{\bullet} \quad (153\ m/z)$$

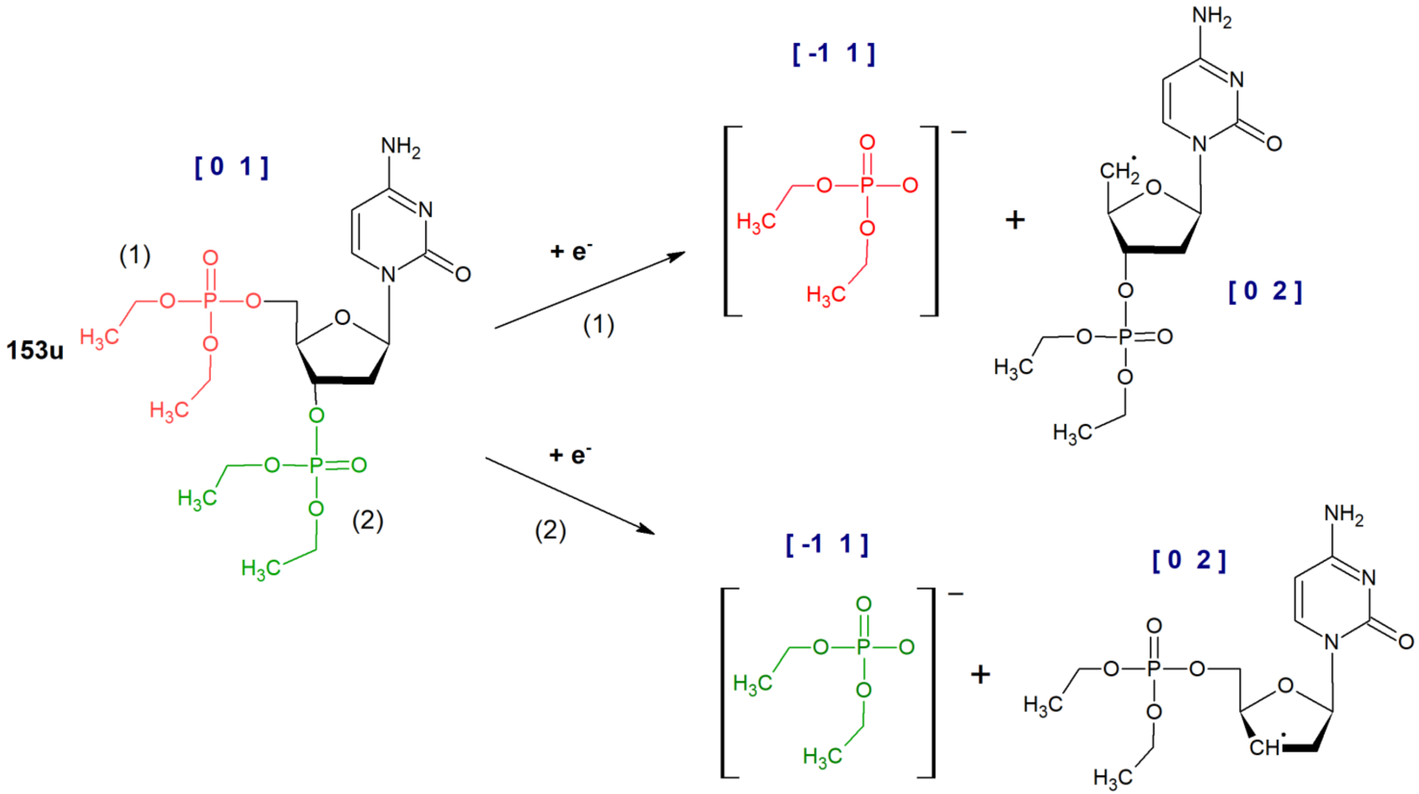

$$\mathrm{tetra-Et-dCDP} + e^- \rightarrow (\mathrm{tetra-Et-dCDP})^{*-} \rightarrow (C_6H_5N_2O)^- + (C_{11}H_{26}NO_9P_2)^{\bullet} \quad (121\ m/z)$$

121u

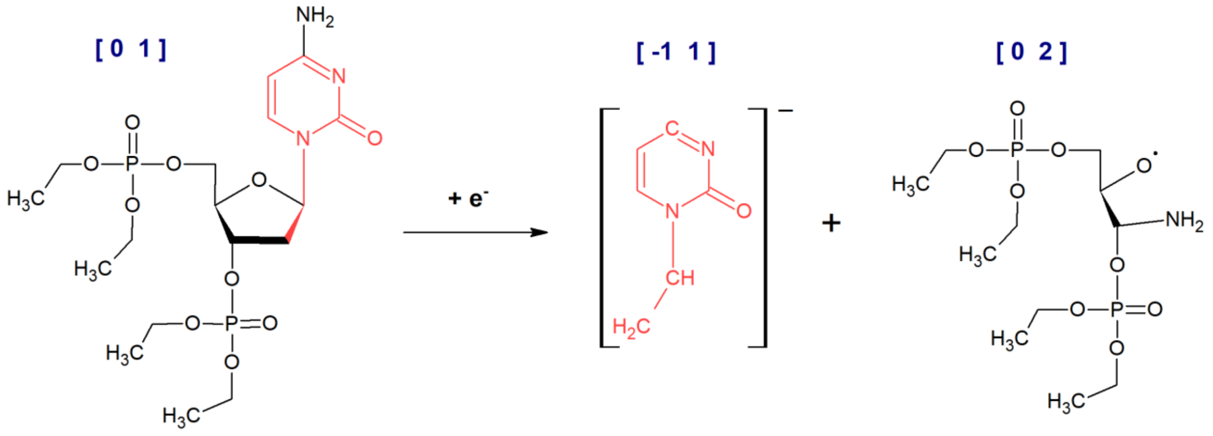

$$\mathrm{tetra-Et-dCDP} + e^- \rightarrow (\mathrm{tetra-Et-dCDP})^{*-} \rightarrow (C_5H_6O_2)^{\bullet-} + \mathrm{cytosine} + C_8H_{20}O_7P_2 \quad (98\ m/z)$$

98u

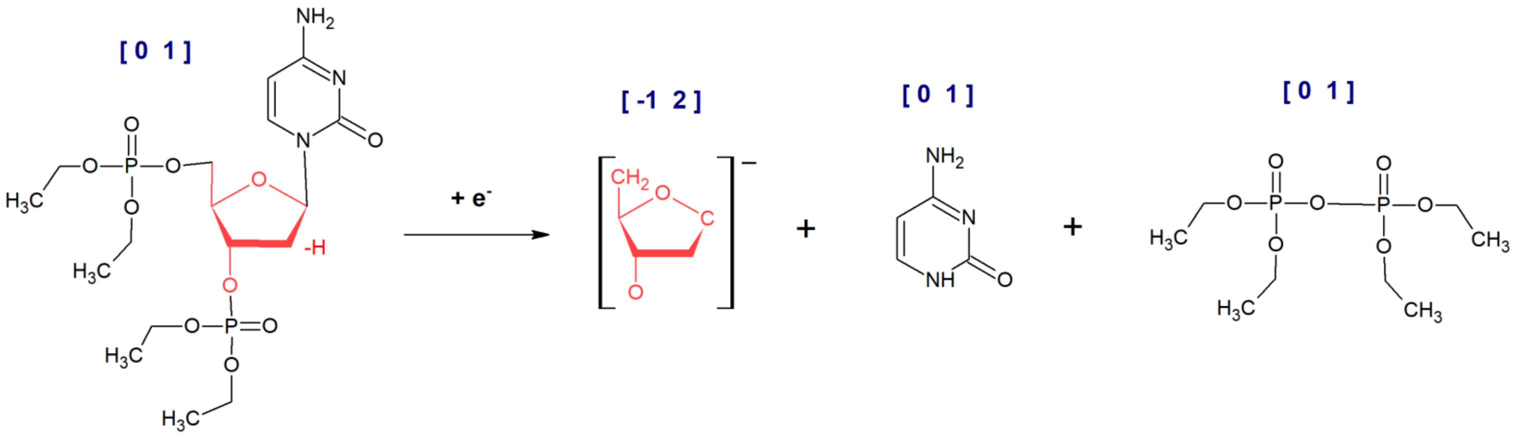

$$\text{tetra}-\text{Et}-\text{dCDP}+e^{-}\rightarrow(\text{tetra}-\text{Et}-\text{dCDP})^{*-}\rightarrow(C_2H_3O_2)^{-}+C_{11}H_{18}N_3O_5P+(C_4H_{10}O_3P)^{\bullet}\qquad(59\ m/z)$$

59u

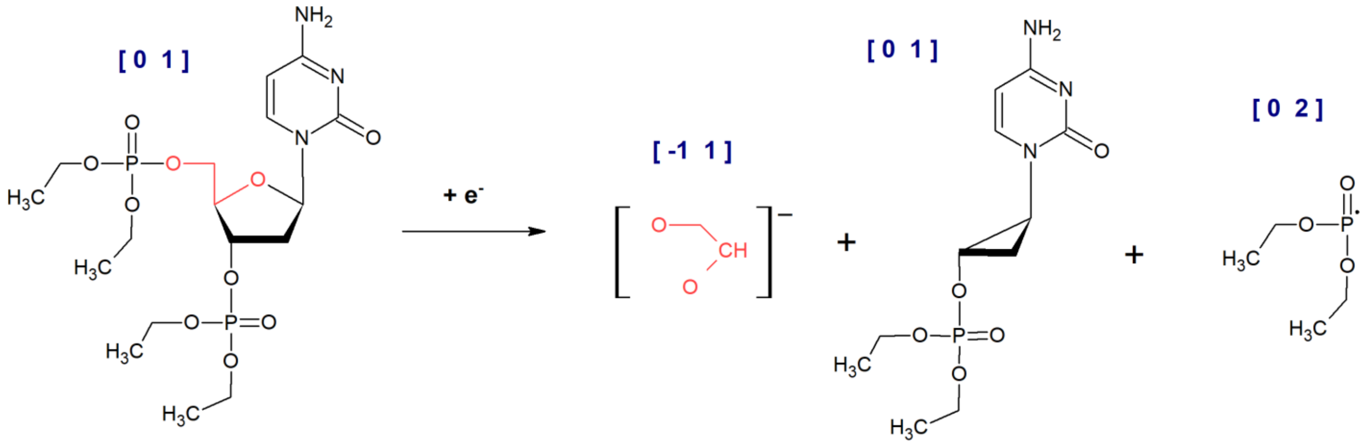

$$\text{tetra}-\text{Et}-\text{dCDP}+e^{-}\rightarrow(\text{tetra}-\text{Et}-\text{dCDP})^{*-}\rightarrow(C_2H_5O)^{-}+(C_{15}H_{26}N_3O_9P_2)^{\bullet} \quad (45\ m/z)$$

45u

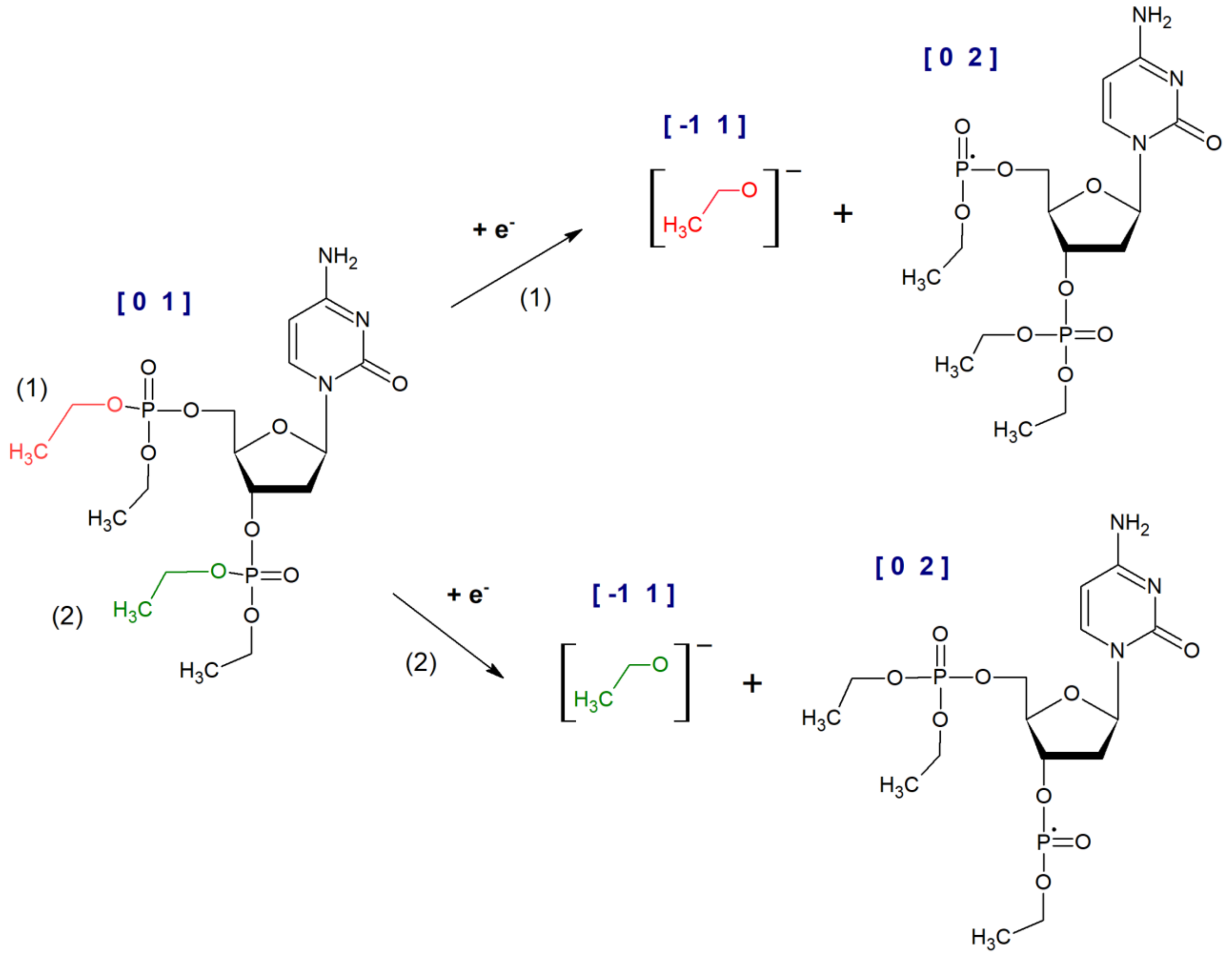

$$\text{tetra} - \text{Et} - \text{dCDP} + e^{-} \rightarrow (\text{tetra} - \text{Et} - \text{dCDP})^{*-} \rightarrow (\text{OH})^{-} + (\text{tetra} - \text{Et} - \text{dCDP} - \text{OH})^{\bullet} \qquad (17\ \text{m/z})$$

17u

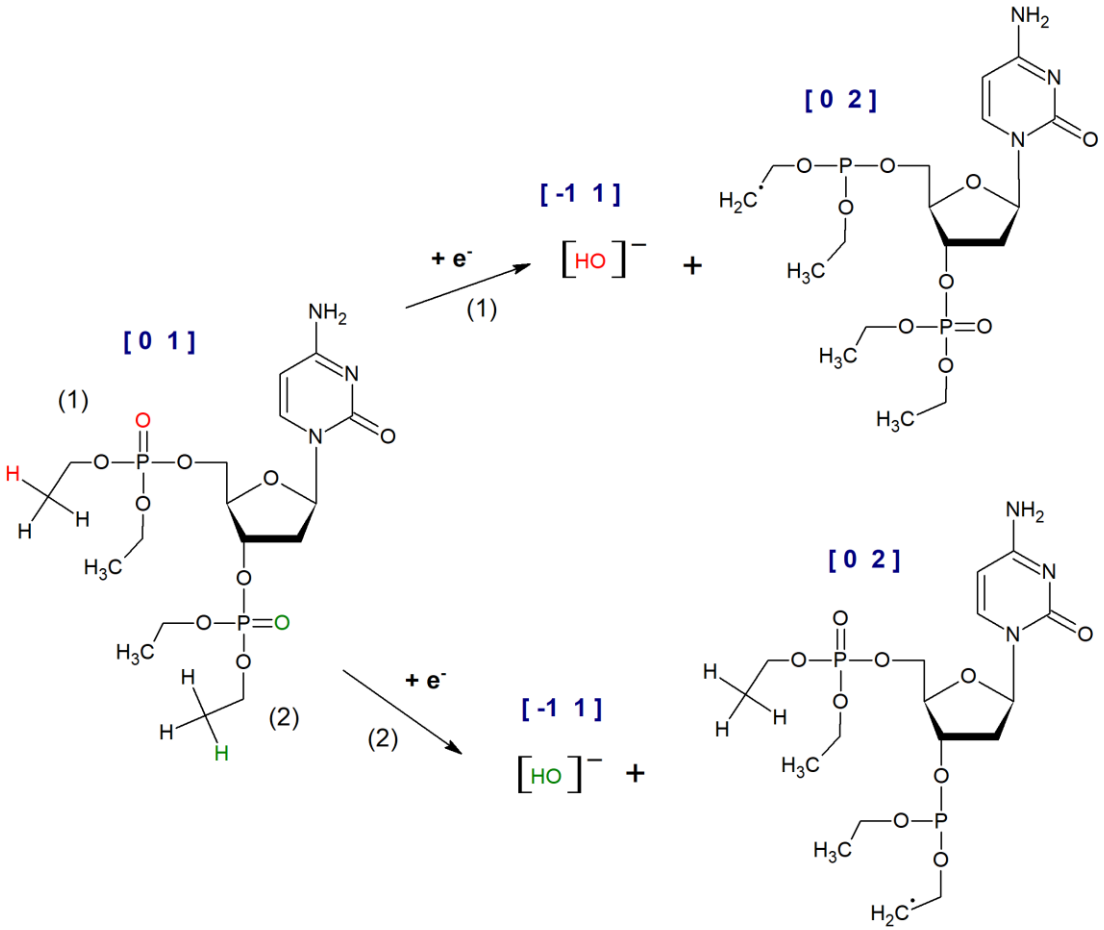

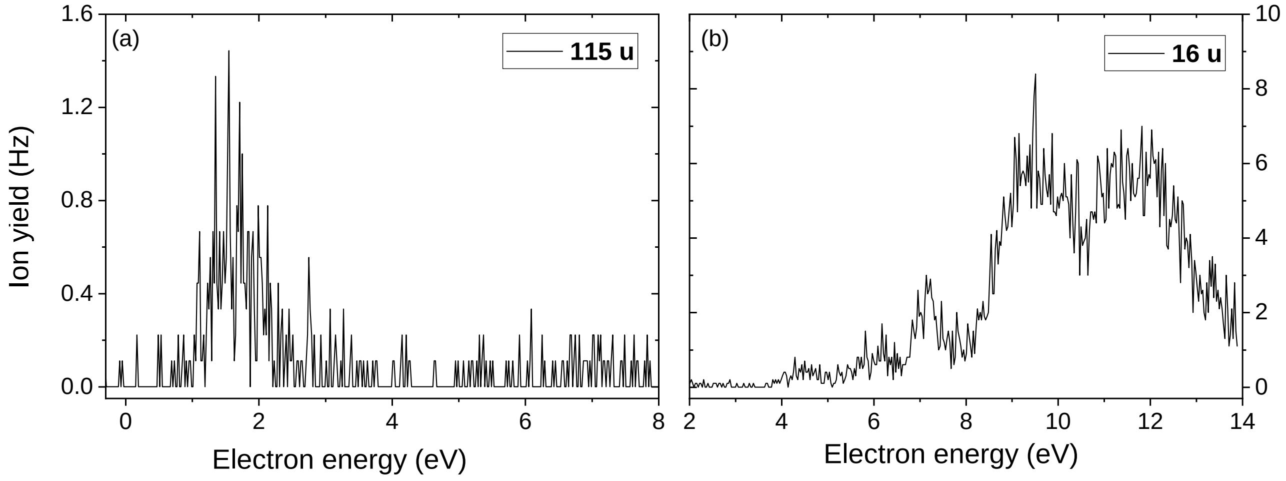

Figure S9. Anion efficiency curves for the formation of the anion with masses (a) 115 u, and (b) 16 u upon electron attachment to the native dCMPH.

Table S3. Summary of fragment anions in terms of mass, structural assignments, peak maxima and thermodynamic thresholds for the native dCMPH. The main dissociation channel marked red. For the other fragment anions, which are much less abundant than the main product, a significant deviation of the experimental threshold and the calculated reaction enthalpy is notable. This deviation can be explained by two possible reasons, (i) the presence of impurities in the experiment, which lead to the formation of these anions or (ii) other reaction pathways in DEA to dCMP lead to the formation of the ion yield at a certain m/z value than the calculated ones. For example, the ion yield at m/z 16 below the calculated lowest threshold of 4.15 eV may be also ascribed to NH_2^- formation not considered in the calculations.

**Mass m/z**	**Anion of dCMPH**	**Maxima of peak positions (eV)**							**Enthalpy (ΔH) [eV]**		
		**1.**	**2.**	**3.**	**4.**	**5.**	**6.**	**7.**	**Exp. 398.15 K**	**Calc. 298.15 K**	**Calc. 398.15 K**
115	$(C_5H_7O_3)^-$	1.63	--	--	--	--	--	--	0.75[a]	3.64 (1)[b]; 5.77 (2)	3.64 (1); 5.77 (2)
**97**	**$(H_2O_4P)^-$**	**0.06**	**0.36**	**1.31**	**2.22**	**3.68**	**5.85**		**~0[a]**	**0.07**	**0.08**
16	O^-	4.45	6.08	7.16	7.76	9.27	11.66	--	3.59	4.68 (1)[c]; 4.15 (2)	4.70 (1); 4.16 (2)

[a] Different experimental parameters: 97 m/z ($p = 1.95 \times 10^{-10}$ atm, T = 388.15 K), 115 m/z ($p = 1.09 \times 10^{-10}$ atm, T = 392.15 K)
[b] threshold (1) – proton transfer from C2' in sugar moiety to oxygen on carbon C5'; (2) no proton transfer.
[c] threshold (1) for oxygen from phosphate group (P=O); (2) for oxygen from cytosine (C2=O).

Molecular and structural equations of possible anion formation pathways (charge and multiplicity marked blue):

$$dCMPH + e^{-} \rightarrow (dCMPH)^{*-} \rightarrow (C_5H_7O_3)^{-} + H_2O_3P^{\bullet} + cytosine \qquad (115\ m/z)$$

115u

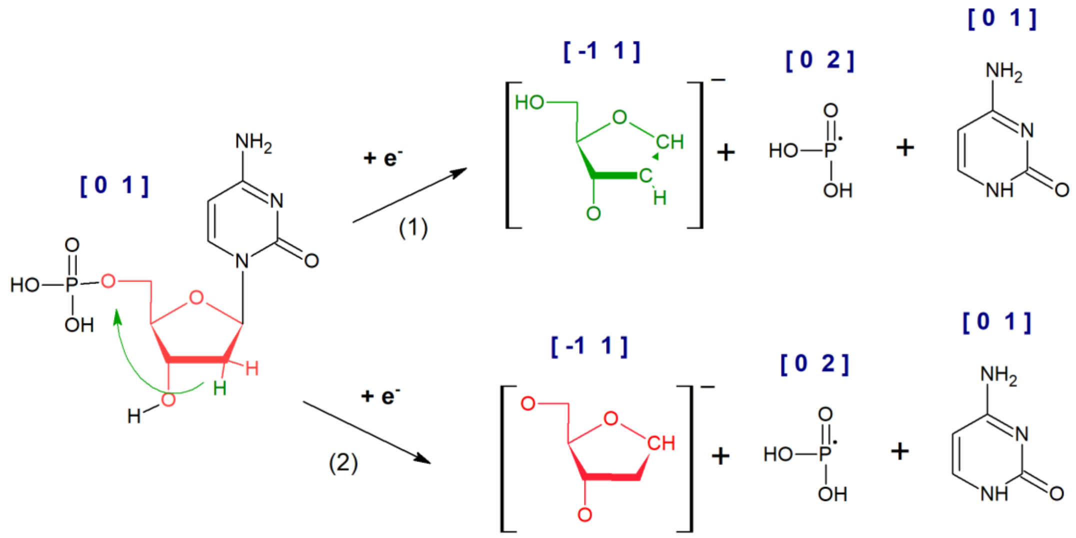

$$dCMPH + e^{-} \rightarrow (dCMPH)^{*-} \rightarrow (H_2O_4P)^{-} + (C_9H_{12}N_3O_3)^{\bullet} \qquad (97\ m/z)$$

97u

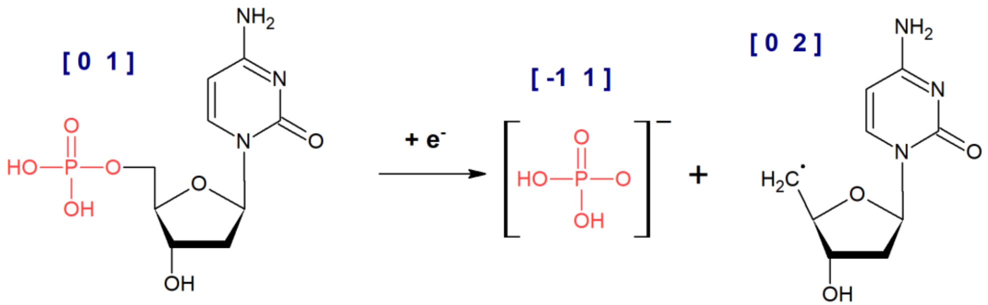

$$\text{dCMPH} + e^- \rightarrow (\text{dCMPH})^{*-} \rightarrow (\text{O})^{\bullet -} + (\text{dCMPH} - \text{O}) \qquad (16\ \text{m/z})$$

16u

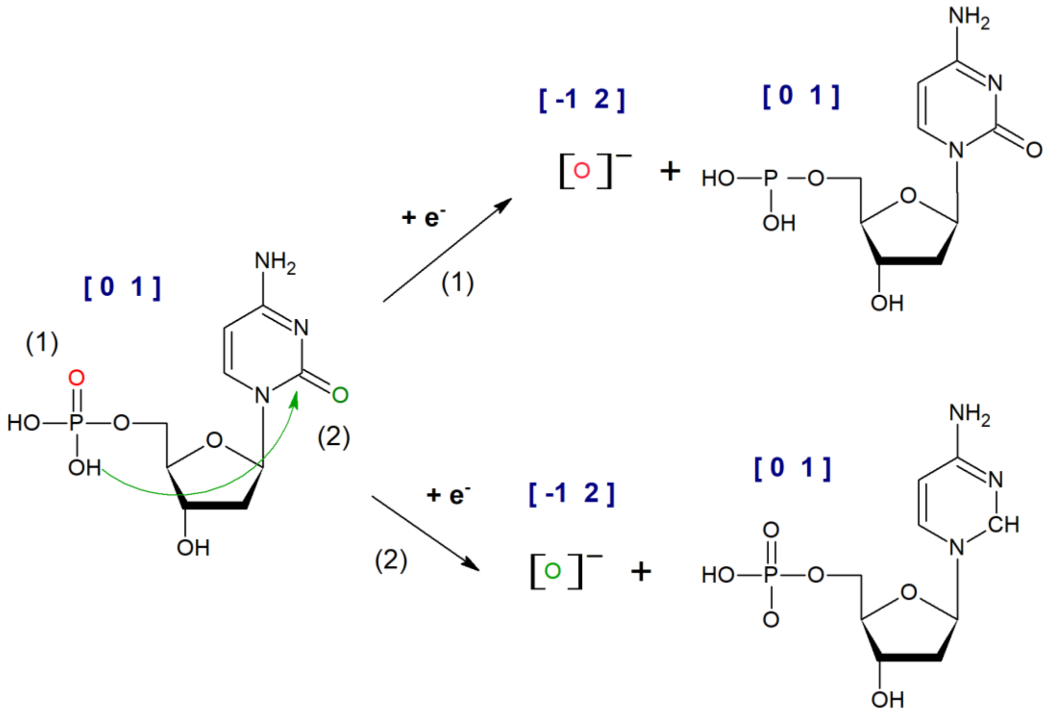

Supplement: Supplementary file 1 — ja3c00591_si_001.pdf [file ja3c00591_si_001.pdf]
